# Engineering grain boundaries at the 2D limit for the hydrogen evolution reaction

**DOI:** 10.1038/s41467-019-13631-2

**Published:** 2020-01-02

**Authors:** Yongmin He, Pengyi Tang, Zhili Hu, Qiyuan He, Chao Zhu, Luqing Wang, Qingsheng Zeng, Prafful Golani, Guanhui Gao, Wei Fu, Zhiqi Huang, Caitian Gao, Juan Xia, Xingli Wang, Xuewen Wang, Chao Zhu, Quentin M. Ramasse, Ao Zhang, Boxing An, Yongzhe Zhang, Sara Martí-Sánchez, Joan Ramon Morante, Liang Wang, Beng Kang Tay, Boris I. Yakobson, Achim Trampert, Hua Zhang, Minghong Wu, Qi Jie Wang, Jordi Arbiol, Zheng Liu

**Affiliations:** 1 School of Materials Science and Engineering, Nanyang Technological University, Singapore, 639798 Singapore; 2 Center for OptoElectronics and Biophotonics, School of Electrical and Electronic Engineering & The Photonics Institute, Nanyang Technological University, Singapore, 639798 Singapore; 3 Catalan Institute of Nanoscience and Nanotechnology (ICN2), CSIC and BIST, Campus UAB, Bellaterra, Barcelona, 08193 Catalonia Spain; 4 Catalonia Institute for Energy Research (IREC), Jardins de les Dones de Negre 1, Sant Adrià del Besòs, Barcelona, 08930 Catalonia Spain; 5 College of Aerospace Engineering, Nanjing University of Aeronautics and Astronautics, Nanjing, 210016 China; 6 Department of Materials Science and NanoEngineering, Rice University, Houston, TX 77005 USA; 7 Paul-Drude-Institut für Festkörperelektronik Leibniz-Institut im Forschungsverbund Berlin Hausvogteiplatz, 5-7, 10117 Berlin, Germany; 8 Centre for Micro-/Nano-electronics (NOVITAS), School of Electrical & Electronic Engineering, Nanyang Technological University, 50 Nanyang Avenue, Singapore, 639798 Singapore; 9 Institute of Fundamental and Frontier Sciences, University of Electronic Science and Technology of China, Chengdu, 610054 China; 10 CNRS-International-NTU-THALES Research Alliance, Nanyang Technological University, Singaproe, 637553 Singapore; 11 Institute of Flexible Electronics, Northwestern Polytechnical University, Xi’an, 710072 China; 12 SuperSTEM Laboratory, SciTech Daresbury Campus, Keckwick Lane, Daresbury, WA44AD UK; 13 School of Chemical and Process Engineering, University of Leeds, Leeds, LS29JT UK; 14 College of Materials Science and Engineering, Beijing University of Technology, Beijing, 100124 People’s Republic of China; 15 School of Environmental and Chemical Engineering, Shanghai University, Shanghai, 200444 China; 16 Department of Chemistry, City University of Hong Kong, Tat Chee Avenue, Kowloon, Hong Kong China; 17 CINTRA CNRS/NTU/THALES, UMI 3288, Research Techno Plaza, Singapore, 637553 Singapore; 18 ICREA, Pg. Lluís Companys 23, Barcelona, 08010 Catalonia Spain; 19 Environmental Chemistry and Materials Centre, Nanyang Environment and Water Research Institute, Singapore, Singapore

**Keywords:** Catalyst synthesis, Electrocatalysis, Two-dimensional materials

## Abstract

Atom-thin transition metal dichalcogenides (TMDs) have emerged as fascinating materials and key structures for electrocatalysis. So far, their edges, dopant heteroatoms and defects have been intensively explored as active sites for the hydrogen evolution reaction (HER) to split water. However, grain boundaries (GBs), a key type of defects in TMDs, have been overlooked due to their low density and large structural variations. Here, we demonstrate the synthesis of wafer-size atom-thin TMD films with an ultra-high-density of GBs, up to ~10^12^ cm^−2^. We propose a climb and drive 0D/2D interaction to explain the underlying growth mechanism. The electrocatalytic activity of the nanograin film is comprehensively examined by micro-electrochemical measurements, showing an excellent hydrogen-evolution performance (onset potential: −25 mV and Tafel slope: 54 mV dec^−1^), thus indicating an intrinsically high activation of the TMD GBs.

## Abstract (web summary)

Transition metal dichalcogenides demonstrate fascinating capabilities for electrocatalytic H_2_ evolution, although the activities vary widely depending on nanomaterial sites available. Here, authors show the grain boundaries of atomically thin MoS_2_ to be especially active sites for H_2_ evolution.

## Introduction

**Fig. 1:**
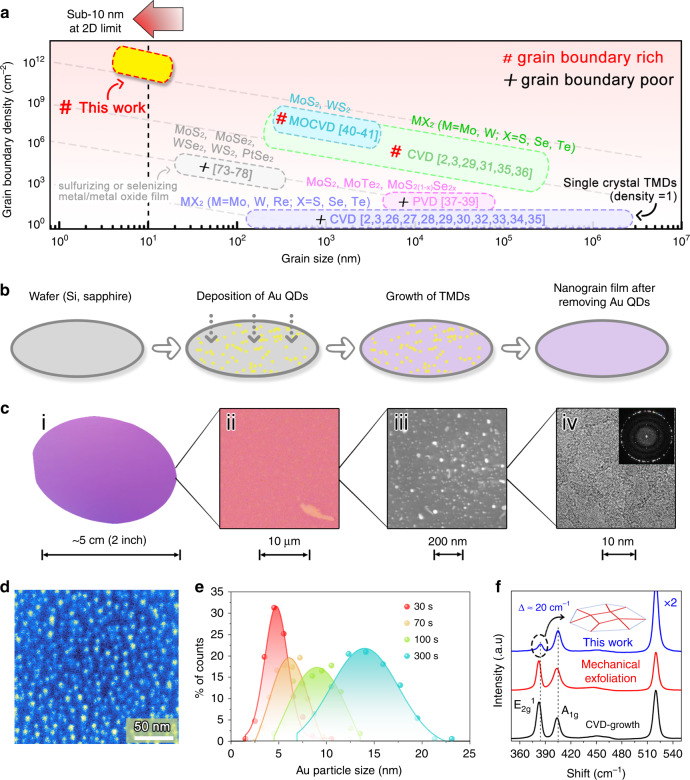
Synthesis of wafer-size TMD nanograin films. **a** Overview of the grain size and density in TMD materials obtained by various fabrication methods, such as CVD^2,3,26–36^, MOCVD^40,41^, PVD^37–39^, top–down syntheses of sulfurization/selenization of metal and metal oxide thin films^73–77^, and thermal decomposition of thiosalts thin films^78^. Although small grains (~20 nm in diameter) were observed in TMDs synthesized by the top–down methods of sulfurizing Mo-based thin films^73,74^, or thermal-decomposition of Mo-thiosalts thin films^78^, the grain density in the film is not very high owing to their interlayer diffusion synthesis mechanism. **b** Schematic of the wafer-scale growth of TMD nanograin films. Ultra-high-density Au quantum dots (QDs) were used to grow the MoS_2_ (as well as WS_2_ atom-thin films: see Supplementary Information). **c** TMD nanograin film from wafer scale to nanoscale, including photograph (i), optical image (ii), SEM image (iii), and HRTEM image (iv). **d** False-colored SEM image of the Au QDs on a SiO_2_/Si substrate, showing an average diameter of 4.8 nm. **e** Statistical distributions of Au QDs obtained from Au films with different deposition times. The evaporation rate is ~0.1 Å s^−1^ in our experiments. **f** Raman spectra acquired from the MoS_2_ film. The difference (*∆* ~20 cm^−1^) between the out-of-plane (A_1g_) and in-plane (E^1^_2g_) Raman modes indicates that the MoS_2_ film is 1–3 layers (1–3 L)^2,44^. Compared to CVD-grown and exfoliated samples, the reduced intensity of the in-plane mode indicates a highly polycrystalline structure for this MoS_2_ film.

Grain boundaries (GBs) are commonly found in atom-thin or so called two-dimensional (2D) polycrystalline materials^1–3^, where they can be described as line defects. They play a key role in shaping the properties and performance of 2D materials in applications as varied as mechanical strengthening^4^, photovoltaics^5,6^, electronics^7–9^, and catalysis^10,11^. Engineering the structure and/or the density of GBs in 2D materials could thus become a promising way to tailor their performance. One particular class of 2D materials, transitional metal dichalcogenides (TMDs) have attracted a great deal of attention for its possible uses in electrocatalytic reactions^12,13^, including the hydrogen evolution reaction (HER)^14,15^. Due to the low cost, earth abundance and good stability of a wide range of TMDs, substantial work has thus been undertaken to improve their electrocatalytic activity^12,13^ by, e.g., exposing their edges^16–19^, doping with heteroatoms^20^, and/or creating and straining structural defects^11,15,19,21,22^. In contrast, less attention has been paid to the role of GBs due to their typically low number density and large structural variations, even though GBs have been predicted to be highly electrocatalytically active^23^. The poor control over the density and structure of GBs stems from the fast gaseous kinetic processes and the multiplicity of chemical phases involved during the growth of TMDs^2,24,25^. To date, the most common methods employed to synthesize atomically thin polycrystalline TMDs include chemical vapor deposition (CVD)^2,3,26–36^, physical vapor deposition (PVD)^37–39^, and metal organic chemical vapor deposition (MOCVD)^40,41^. Films grown using these techniques usually exhibit grain sizes ranging from hundreds of nanometers to several millimeters, resulting in a low GB density, as summarized in Fig. 1a (See the statistical method in Supplementary Fig. 1).

Here, we fabricate wafer-size atomically thin TMD films with sub-10 nm grains by means of Au-quantum-dots (QDs)-assisted vapor-phase growth, demonstrating an ultra-high-density of GBs (up to ~10^12^ cm^−2^). The quality of the films was examined by high-resolution transmission electron microscopy (HRTEM), aberration-corrected scanning transmission electron microscopy (STEM), scanning electron microscopy (SEM), and Raman spectroscopy. Experimental evidence as well as phase-field simulations demonstrate that the Au QDs regulate the formation of the TMD grains. We then investigated the catalytic activity of these nanograin films by a four-electrode micro-electrochemical cell for hydrogen evolution. An excellent performance (−25 mV and 54 mV dec^−1^ for the onset potential and the Tafel slope) was obtained for our MoS_2_ nanograin films, indicating a good intrinsic activation of the GB-rich 2D basal plane.

## Results

### Controlled growth of the TMD nanograin film

The main obstacles that hinder the vapor growth of TMDs at the 2D limit to grain sizes typically <10 nm consist of the precise control of the nucleation sites and the growing rate of the grains. These can be addressed by using a high-density of Au QD seeds and a low mass flow rate of the vapor sources. Figure 1b illustrates the wafer-scale growth process (see also a full description of the growth setup in Supplementary Fig. 2). A wafer-scale Au QD layer was fabricated on 2-inch sapphire or SiO_2_/Si substrates and used to subsequently grow the atom-thin MoS_2_ films. Figure 1c shows the geometries of the as-grown wafer-size film from the centimeter to the nanometer scale. The SEM image (Fig. 1d) shows the as-prepared Au QDs with an ultra-high density up to ~2 × 10^12^ cm^−2^ and average diameter down to ~4.8 nm, which was achieved by heating a thin Au film on sapphire or SiO_2_/Si substrates at a high temperature. We attribute the formation process of these Au QDs to a solid-state dewetting behavior at the interface between the SiO_2_ (or Al_2_O_3_) and Au^42,43^ (Supplementary Fig. 3). The initial deposition time of the Au films prior to heating determines the density and the size of the final Au QD structures: a shorter deposition time results generally in a smaller size and a higher density of Au QDs, as shown in Fig. 1e and Supplementary Fig. 4. The MoS_2_ film is then grown using a vapor-phase growth technique (see further experimental details in Method section), after which Au QDs can be removed from as-grown films, by using KI/I_2_ etchant at room temperature. The successful removal of the Au QDs after the etching treatment was assessed by X-ray photoelectron spectroscopy and STEM imaging in Supplementary Figs. 5–7, showing only minimal amounts of residual Au present. The resulting MoS_2_ films were examined by Raman spectroscopy, as shown in Fig. 1f. The Raman spectroscopy measurements indicate that the MoS_2_ film is 1–3 layers thick (1–3 L). A weak in-plane mode (E_2g_^1^ at 385 cm^−1^) was observed in our 1–3 L film, with an intensity ratio with the out-of-plane mode A_1g_ (at 405 cm^−1^), $I_{E_{2g}^1}/I_{A_{1g}}$ (~0.28), significantly smaller than the ratios typically measured on the CVD-grown (~1.89) and exfoliated (~1.57) MoS_2_ films in our experiment, suggesting a highly polycrystalline structure in this film (see the details in Supplementary Table 1 and Supplementary Note 1).

Au QDs regulate the formation of TMD grains in two ways. The first way is that Au QDs can facilitate the TMD nucleation at the initial stage of the TMD growth. The melting temperature of Au will dramatically decrease as its size decreases to several nanometers, as shown in Supplementary Fig. 8. As a result, at the growth temperature of TMDs (650–800 °C), Au QDs tend to be in the liquid phase due to their small size. Such liquid Au droplets can facilitate the formation of the TMD nucleation sites during the growth process. The second way is that the Au QDs can confine the size of the TMD domains below 10 nm. Our deposition method can produce well-dispersed Au QDs with a very high number density, with average spacing between QDs down to a few nanometers. As a result, the formation of the TMD grains will be constrained at this length scale.

### Atomic structure of the TMD nanograin films

**Fig. 2:**
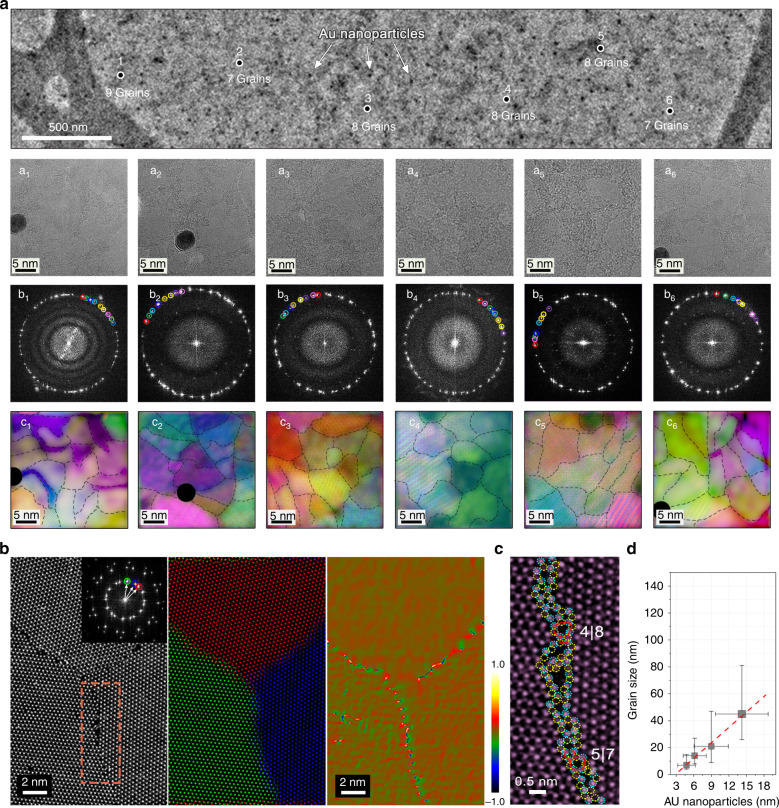
Atomic structure of the TMD nanograin film. **a** TEM image of the 1–3 L MoS_2_ nanograin film, showing a uniform and continuous atom-thin layer with Au nanoparticles (dark spots). Top panel (a1–a6): HRTEM images from regions 1–6. Middle panel (b1–b6): Fourier transforms (FFTs) of regions 1–6 from the corresponding TEM image. Bottom panel (c1–c6): false-colored frequency filtered (IFFTs) images of the same regions. Grains with different orientations give rise to distinct sets of rotated spot patterns in the FFTs, as indicated by colored circles in the middle panel (b1–b6); in turn, the localization of the grains can be visualized in real space by color coding the corresponding contribution to the IFFT of a given set of diffraction spots, and overlaying the IFFTs into a composite colored image (in bilayer regions, the colors are combined). In order to better visualize the different grains, the grains are highlighted in the IFFT images with black-dashed lines. **b** HAADF STEM investigation of MoS_2_ grains. Left: HAADF STEM image showing the GBs between three MoS_2_ grains. Inset on the left figure: Fourier transform of the image showing three distinct sets of MoS_2_ diffraction patterns with rotation angles of 11.6° and 25.6°. Middle: composite color-coded IFFT image. Right: dilatation map of **b** after applying GPA routine to the monolayer MoS_2_. **c** Higher magnification atomic resolution HAADF STEM image of the brown-dashed line marked region in **b**, showing the detailed atomic structure of the GB, which is composed of 5|7 and 4|8 rings. Mo atoms are marked with indigo dotted circles and S atoms marked with yellow dotted circles. **d** Grain size dependence on Au nanoparticle size (average diameter) in the MoS_2_ nanograin film. The error bars of Au nanoparticles are extracted from the full-width at half maximum in Fig. 1e.

We investigated the atomic structure of the TMD nanograin films using transmission electron microscopy (TEM). Figure 2a shows an overview TEM image of a uniform and continuous MoS_2_ film suspended on a Cu-supported lacey carbon TEM grid. This region is representative of the tens of samples examined, and the MoS_2_ film comprises polycrystalline patches with regions of 1–3 L MoS_2_ (consistent with the measured Raman frequencies^44^). To evaluate the grain distribution, we randomly chose six regions of the film, labeled 1–6 on Fig. 2a, for further HRTEM imaging. The original HRTEM images from these six regions, corresponding Fourier transforms (FFTs) and frequency filtered images (IFFTs)^45^ are shown in the top (a1–a6), middle (b1–b6), and bottom panels (c1–c6) of Fig. 2a, respectively. To aid the visualization of the different grains, black-dashed lines are added along the edges of the grains in the bottom panel (c1–c6). From these images, we can identify 8–10 distinct MoS_2_ grains in a ~700 nm^2^ region, suggesting an ultra-high grain density (up to ~10^12^ cm^−2^ in this representative patch, consistent with the initial estimate derived from the Au QD number density) in our sample. Accordingly, the average diameter of the grains is <10 nm, with some observed grains smaller than 5 nm in diameter. To our knowledge, this is the smallest grain size obtained to date in materials at the 2D limit^17,46,47^.

We then employed atomic-resolution high-angle annular dark-field (HAADF) imaging in the STEM to examine the atomic structure of the GBs on the as-grown nanograin films, representative examples of which are shown in Fig. 2b, c and Supplementary Fig. 9. The inspection of dozens of boundary locations systematically found that nanograins in our films are stitched by GBs. The atomic structure of the GBs varied significantly (great care was taken to exclude any structure modification arising from beam damage, see Experimental section for details). Structures comprising a combination of 5|7 and 6|8 rings were however dominant in our nanograin films and are illustrated in Fig. 2c. A small number of 8|4|4 structures were found, but in contrast, 12|4 rings were never observed in “pristine” GBs (i.e., before any substantial exposure to the electron beam). Their atomic structures in MoS_2_ are schematically illustrated in Supplementary Fig. 10.

Although the nature of atomic-resolution microscopy precludes large-scale statistical studies, we investigated as many different regions in a MoS_2_ nanograin film as practically possible. Six further regions over a large range were carefully examined with atomically resolved HAADF–STEM imaging (Supplementary Fig. 11), and 3 to 5 GBs can be observed in a 400 nm^2^ area, confirming the ultra-high-density of GBs observed in STEM (consistent with the ~10^12^ cm^−2^ suggested by the Au QD density). Moreover, tens of samples with different sizes of Au QDs were grown: the grain size measured in the resulting MoS_2_ films exhibits a clear linear relationship with the QD size, as shown in Fig. 2d. This provides a strong proof that the TMD grain size can be carefully controlled by the Au QD substrate. Remarkably, our method can be also extended to other TMD materials such as WS_2_ (Supplementary Fig. 12 summarizes similar results to those described above, using WS_2_ instead of MoS_2_), making it as a universal approach for the wafer-size synthesis of atom-thin films with sub-10 nm grains.

### Growth mechanism of the TMD nanograin film

**Fig. 3:**
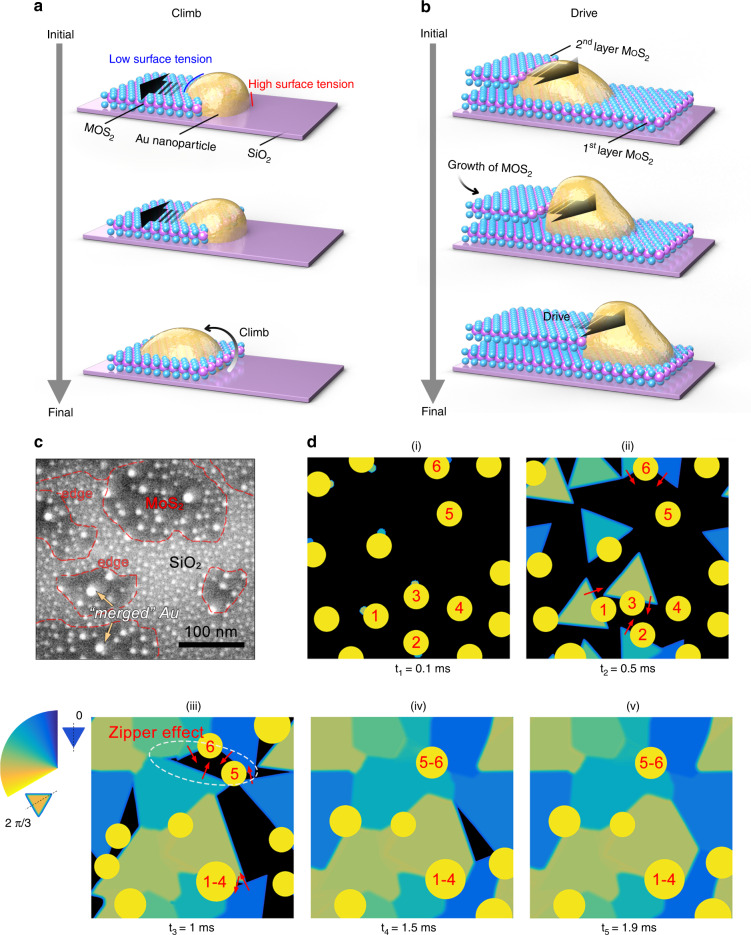
Proposed self-limiting climb and drive growth mechanism of the TMD nanograin films. **a** Schematic of the climb stage: the Au QDs droplets tend to climb onto the MoS_2_ monolayer from the SiO_2_ substrate due to the surface tension difference. **b** Schematic of the drive stage: the growth of a second MoS_2_ layer tends to push the Au QDs along the growth direction. Most of the Au QDs will be stabilized at the grain boundaries in order to minimize their surface energy. **c** SEM image showing the distribution of the Au QDs on the edges of growing MoS_2_ layers (marked by the red-dashed line). Au QDs are usually larger on the MoS_2_ layers (indicated by blue arrows) than those observed on the SiO_2_ substrate. **d** Real-time phase-field simulation showing the formation of the MoS_2_ nanograin film. The Au QDs numbered 1–4 show that the growth of the MoS_2_ layer will push Au QDs toward the grain boundaries. Au QDs numbered 5–6 show a zipper effect to suture the neighboring grains together. The movie can be found in Supplementary Movie 1 (Real-time phase-field simulation of the growth process).

A climb and drive zero-dimensional (0D)/2D interaction is proposed to explain the Au QDs-assisted-growth mechanism of the TMD nanograins at the atomic limit, which is supported by real-time phase-field simulations and experimental evidence. The phase-field simulation of the climb process shows that, once the Au QDs encounter a MoS_2_ edge at the growth front, they migrate swiftly from the SiO_2_ surface onto the MoS_2_ surface (Fig. 3a). This behavior is mainly attributed to a smaller wetting angle of the Au QDs droplet (Au is in the liquid phase at the growth temperature) on MoS_2_ than that on the SiO_2_ substrate^48^ (Supplementary Fig. 13). This phenomenon is also experimentally confirmed by cross-sectional TEM, as shown in Supplementary Fig. 14, where nearly all the Au QDs sit on the surface of the MoS_2_. Subsequently, the phase-field simulation suggests the formation of a second MoS_2_ layer will drive the Au QD droplets along its growth direction (Fig. 3b). A number of the Au QDs may thus coalesce and form bigger droplets during the driving process. This is supported by our experimental evidence: the size of the Au QDs on the MoS_2_ is usually larger than those on SiO_2_, and some of them sit at the edge of the MoS_2_ sheet, as illustrated in Fig. 3c and Supplementary Fig. 15a–b. This drive process will be immobilized when the Au QDs reach a critical size (as a result of the Au returning to the solid phase). This would be consistent with experimental observations that larger Au particles seen in the minority multilayer regions of the nanograin film appear to be encapsulated by the MoS_2_ film, suggesting they were immobilized after reaching a critical size, leading the MoS_2_ to grow over them rather than driving them forward (see Supplementary Figs. 15c, d and 16).

Figure 3d shows successive snapshots that illustrate the growth of TMD nanograins films via a phase-field simulation (also see Supplementary Movie 1). The Au QDs serve as nucleation sites to form the first MoS_2_ layer (Fig. 3d–i). The growth of a second MoS_2_ layer will then drive the Au QDs. Some of the Au QDs will be immobilized at the GBs (Fig. 3d ii and iii, number 1–4). More interestingly, we found that neighboring grains could be sutured together through a zipper effect (Fig. 3d iii-v, number 5 and 6, see Method and Supplementary Fig. 17). It is important to emphasize that the growth model for the TMD nanograin films we describe here is driven by a 0D/2D interaction at the atomic limit, which is distinct from the conventional metal-film-assisted growth of 2D materials (2D/2D)^29,35^, or from the metal-nanoparticle-assisted growth of nanowires (0D/1D)^49^.

### Hydrogen production of the TMD nanograin films

**Fig. 4:**
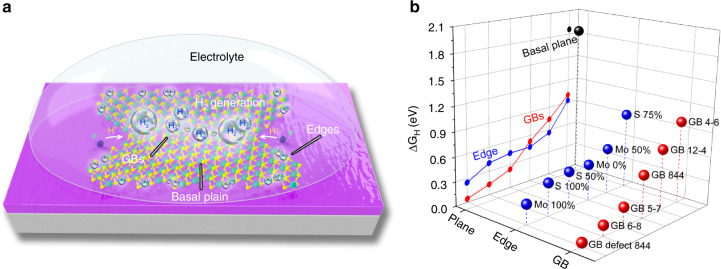
Theoretical calculations of the catalytic sites in MoS_2_ for HER. **a** Schematic of MoS_2_ with an illustration of the main types of catalytically active sites for the hydrogen evolution reaction (HER) including: basal plane atomic sites, edges, and GBs. **b** corresponding *∆G*_H_ of various types of catalytically active sites in MoS_2_ catalysts. Some types of GBs (e.g., 844, 6–8, and 5–7) show better electrocatalytic activity than edges.

We first performed first-principle calculations^50^ to examine all of the HER catalytic active sites in model MoS_2_ geometries (Fig. 4a), including the basal plane sites, edges with different passivation (Mo or S)^51^, and GBs with different atomic structural configurations (5|7, 6|8, 4|6, 12|4, and 8|4|4 rings). The calculated hydrogen adsorption free energies (*∆G*_H_)^52^ of various atomic structures (Supplementary Fig. 18) are shown in Fig. 4b and Supplementary Table 2. The MoS_2_ basal plane has a *∆G*_H_ as high as 1.79 eV^15^, indicating a HER-inert surface. For edges and GBs, we compare the activities of most of the experimentally observed structures, e.g., 5|7 (0.132 eV), 6|8 (−0.237 eV), and 8|4|4 (0.52 eV, −0.044 for defect) ring combinations,^2,53^ 50% S passivated Mo edge (0.561 eV), and 50% passivated S edge (0.446 eV)^53,54^. It can be seen that GBs indeed show comparable or even better activity than the edges in general, suggesting GBs are promising candidates as high-efficient catalyst sites. In addition, we note that some edges are not further considered here owing to their instability in air; for instance, the otherwise energetically favorable Mo edge without passivation of S atoms (−0.446 eV).

**Fig. 5:**
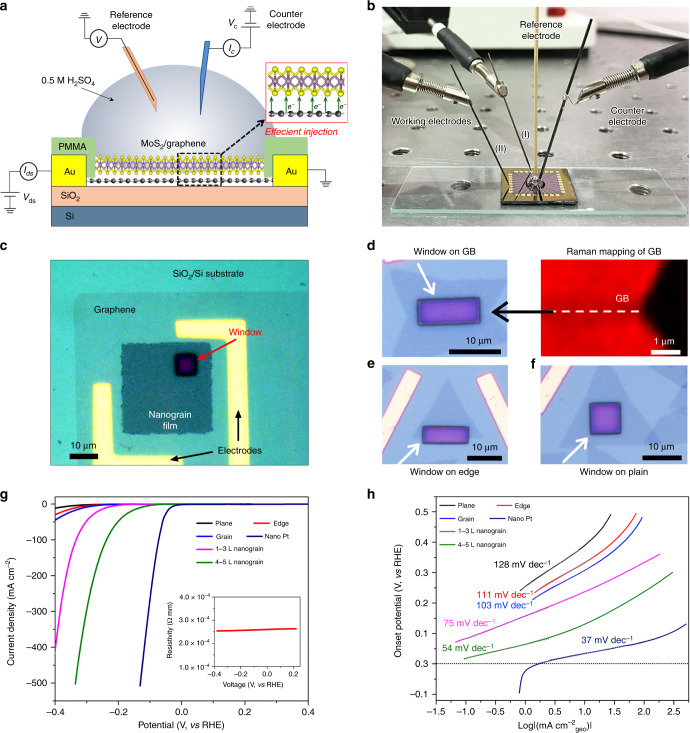
Micro-electrochemical cell-based HER activity of a MoS_2_ nanograin film. **a** Schematic of the micro-electrochemical cell for HER measurements, where graphene serves as a vertical electron injector. **b** Photograph of the micro-electrochemical cell. **c** Optical images of the MoS_2_ nanograin device, consisting of a PMMA reaction window, a MoS_2_ nanograin film, a graphene supporting layer, and a SiO_2_/Si substrate from top to bottom. **d**–**f** Optical images of the MoS_2_ devices with a single GB (**d**), a single edge (**e**), and basal plane (**f**), respectively. In these devices, the HER process occurs at the exposed widows on the PMMA passivation film as indicated by the white arrow. **g**, **h** Polarization curves of the current density (**g**) and the corresponding Tafel plots (**h**) of the MoS_2_ devices. The window size is ~80 μm^2^ for each device.

We then developed a micro-electrochemical cell to investigate the HER activity of our sub-10 nm nanograin films. Furthermore, the HER activity of model single-GBs (mirror GB)^3,11^, single-edge (Supplementary Fig. 19)^53^, and basal plane structures in MoS_2_ samples from CVD method^1^ were also examined for comparison. Figure 5a illustrates the micro-cell’s structure. Figure 5b shows a picture of the micro-cell and electrodes configuration where a graphite counter electrode and a micro-reference electrode were used. In our experiment, a vertical MoS_2_/graphene heterostructure was designed, in which graphene played two important roles: one is to efficiently inject electrons to MoS_2_. Such a strategy has already been widely adopted in TMD-based semiconductor devices^55,56^, while the other is to provide a fair comparison by eliminating the contact resistance variations of the individual MoS_2_ electrodes onto the substrate. Previous work^56,57^ suggested the formation of an inconsistent contact barrier between the MoS_2_ and the Au electrode, owing to their complex metal–semiconductor interface leading to effects such as Fermi pinning, creation of alloy structures, etc. The formation of such a contact barrier was also studied by phase engineering^21^ and field-effect gating^58^ in the HER process. Considering the important role of this graphene supporting layer, we introduced one more working electrode in the micro-electrochemical cell (four-electrode micro-electrochemical cell^59,60^, see circuit diagram in Supplementary Fig. 20) to monitor in situ its conductance. The device fabrication of the MoS_2_/graphene heterostructure is detailed in Supplementary Figs. 21 and 22, and Supplementary Notes 2 and 3. The heterostructure was also characterized by Raman spectroscopy (Supplementary Fig. 23). Figure 5c–f shows the optical images obtained from the typical devices fabricated with a MoS_2_ nanograin film, a single-GB model structure (the presence of a GB was confirmed by Raman mapping, as illustrated on Fig. 5d, right panel), a single edge, and a basal plane, respectively. In these optical images, only the exposed MoS_2_ in the reaction window contributes to the electrocatalytic reaction, as indicated by the white arrows. The rest of the areas are covered by poly(methylmethacrylate) (PMMA), and the graphene supporting layer (Supplementary Fig. 24) are also electrochemically inert.

Figure 5g, h shows the polarization curves and the corresponding Tafel slopes in a 0.5 M H_2_SO_4_ solution and provide several interesting observations. First, the resistivity of the graphene supporting layer is found to be as low as 10^−4^ Ω mm during the HER (see the inset in Fig. 5g), indicating the excellent current injection performance of the graphene layer. Secondly, the single-GB device delivers a better activity than the single-edge device (see also the measurement of the active length per unit area of the GB in Supplementary Fig. 25), and both are superior to the basal plane device, echoing our theoretical calculation results shown above. More importantly, our MoS_2_ nanograin film shows a remarkable HER performance: −25 mV and 54 mV dec^−1^ for the onset potential and the Tafel slope, respectively.

**Fig. 6:**
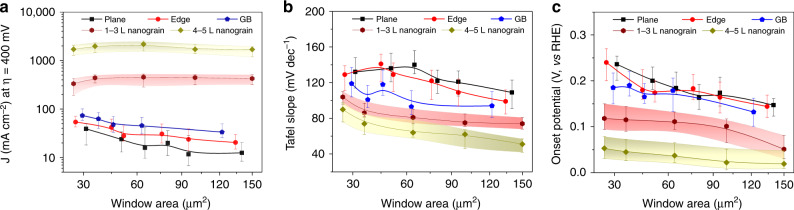
Comparison of the HER performances in a size-controlled micro-electrochemical cell. **a**–**c** Current density (**a**), Tafel plots (**b**), and onset potentials (**c**) for the MoS_2_ devices with serial window sizes from 25–150 μm^2^. The nanograin films show higher current density, lower Tafel slope, and onset potential compared to the basal plane, single edge, and single GB.

In order to evaluate the HER performance of the TMD nanograin film accurately, we have fabricated hundreds of devices and tested them in a size-controlled micro-electrochemical cell. The HER data, including current density, Tafel slope, and onset potential of the nanograin film and other types of electrostatically active sites are presented in Fig. 6a–c and Supplementary Fig. 26. The window size is controlled from 25 to 150 μm^2^. Our results demonstrate the excellent HER performance of the nanograin film. The HER current density of the nanograin film is up to ~1000 mA cm^−^^2^ while the Tafel slope and onset potential are down to ~50 mV dec^−1^ and ~−25 mV, thus exhibiting a performance superior to devices using the basal plane of the CVD film, the single edge and the single GB. This suggests that sites on the otherwise HER-inert basal plane of the MoS_2_ have been activated by the presence of GBs. The obtained HER performance is also comparable to the performance of strained sulfur vacancies^15^ or the metastable 1T-phase^61^, which are arguably more challenging to realize experimentally.

Finally, as a comparison, we have conducted conventional macro-cell measurements on a MoS_2_ nanograin film on a glassy carbon electrode, as shown in Supplementary Figs. 27–32 and Supplementary Notes 4 and 5. A remarkable HER performance (−44 mV and 55 mV dec^−1^ for the onset potential and the Tafel slope, respectively) of the MoS_2_ nanograin film is observed (Supplementary Fig. 28), which is consistent with the results obtained in the micro-cell. It also shows an excellent long-term stability for HER, as shown in Supplementary Fig. 29. It is worth mentioning that Au single-atom exists in our MoS_2_ nanograin film and may contribute to the overall HER performance. However, based on the structure (Supplementary Fig. 6) and extremely low content (Supplementary Fig. 7) of Au single atoms as well as their low HER-activity (Supplementary Figs. 33 and 34, and Supplementary Note 6), we conclude that the residual Au is not the main contributor of the good HER activity in MoS_2_ nanograin film. The measured performance should be mainly from GB due to its superior activity and ultra-high density. As a proof-of-concept practical application, hydrogen production on a wafer-size (2 inch) MoS_2_ nanograin film was also demonstrated (see Supplementary Movie 2). A large amount of H_2_ bubbles could be produced during the reaction, demonstrating the tantalizing potential for practical application of GBs-engineered catalysts for hydrogen production.

## Discussion

In summary, we have engineered wafer-size ultra-high-density GBs at the 2D limit in TMD films. Phase-field simulations reveal a climb and drive 0D/2D-interaction-based growth mechanism. As a proof-of-concept application in electrocatalysis, devices based on our TMD nanograin films deliver a superior hydrogen evolution performance (onset potential: −25 mV and Tafel slope: 54 mV dec^−1^), indicating an intrinsic activation of the GB-rich 2D plane. Beyond electrocatalysis, the nanograin films may provide further diverse potential applications, such as in resistance-memory devices, flexible devices, or for use as mechanical films and molecular sieving films.

## Methods

### Growth of water-size MoS_2_ and WS_2_ nanograin films

The first step consists in depositing Au layers of various thicknesses on clean 2-inch sapphire or SiO_2_/Si wafers by e-beam evaporation at the rate of 0.1 Å s^−1^; these are used as the growth substrate for the nanograin films. Subsequently, Au-coated wafers are introduced into a CVD apparatus, which is depicted in detail in Supplementary Fig. 2. MoO_3_ or WO_3_ powders were loaded in an aluminum oxide boat and used as sources. Sulfur powder is placed in a second aluminum oxide boat upstream of the MoO_3_ or WO_3_ in the outer tube. In a third step, Ar (flow 100 sccm) and Ar/water vapor (flow 50 sccm) are introduced as carrier gases for the growth in the inner and outer tubes, respectively. The growth temperature was kept at 780 °C in low-vacuum conditions of 1–10 kPa; the S powder is separately kept at ~160 °C. Adjusting the growth time is used to control the thickness of films. For example, Condition (I): 3–5 min of the growth time for 1–3 layers (1–3L); Condition (II): 5–10 min of the growth time for 4–5 layers (4-5 L). The growth parameters for WS_2_ nanograin films are similar to those used for MoS_2_, except for a higher growth temperature (900 °C). Finally, Au nanoparticles can be completely removed from as-grown nanograin MoS_2_ or WS_2_ films by using a KI/I_2_ etcher for 40–60 min at room temperature (see Supplementary Figs. 5 and 6).

Special care needs to be taken on two important points: (i) the Au QDs will form once the temperature is above 300 °C, due to the solid-state dewetting behavior^42,43,62^ at the interface of SiO_2_ (Al_2_O_3_) and Au (see Supplementary Fig. 3). Prior to the TMD growth, the substrate therefore stabilizes at this temperature to ensure the presence of Au QDs. (ii) Small amounts of water vapor are used to prevent the Mo or W sources from complete sulfurization before they reach the growth wafer, thus enabling a steady supply of sources during growth.

### Theoretical calculations of ∆G_H_

The structural optimizations were carried out by adopting the generalized gradient approximation (GGA) with the Perdew–Burke–Ernzerhof (PBE) exchange-correlation functional, along with projector-augmented wave (PAW) potentials. The electronic wave functions were expanded in a plane wave basis set with a kinetic energy cutoff of 300 eV. For the Brillouin zone integration, 1 × 5 × 1 Monkhorst–Pack *k*-point meshes were used. The energy convergence criterion for the electronic wavefunction was set at 10^−5^ eV. A vacuum distance of about 10 Å, both laterally between MoS_2_ ribbons and vertically between layers, was chosen when constructing the supercell to minimize possible spurious interaction between ribbons due to the periodic boundary conditions.

The hydrogen adsorption energy is defined as:

1 $$\Delta E_{\mathrm{H}} = E_{({\mathrm{MoS}_{2}} + {\mathrm{H}})} - E_{({\mathrm{MoS}_{2}})} - {^1}/{_2}E_{({\mathrm{H}_{2}})}$$

where $E_{({\mathrm{MoS}_{2}} + {\mathrm{H}})}$ is the energy of the MoS_2_ system with a H atom adsorbed, $E_{({\mathrm{MoS}_{2}})}$ is that of the MoS_2_ system without adsorbed H, and $E_{({\mathrm{H}_{2}})}$ is that of gas phase H_2_ molecule.

The hydrogen adsorption free energy $\Delta G_{\mathrm{H}}$ was defined as:

2 $$\Delta G_{\mathrm{H}} = \Delta E_{\mathrm{H}} + \Delta E_{{\mathrm{ZPE}}} - T\Delta S_{\mathrm{H}}$$

where Δ*E*_*H*_ is the hydrogen adsorption energy, Δ*E*_ZPE_ is the zero-point energy difference, *T* is the temperature, and Δ*S*_H_ is the entropy difference for hydrogen between the adsorbed state and the gas phase. The entropy of hydrogen adsorption is calculated as $\Delta S_{\mathrm{H}} = 1/2S_{{\mathrm{H}_{2}}}$, where $S_{{\mathrm{H}_{2}}}$ is the entropy of hydrogen molecule in the gas phase at standard conditions. We have calculated the vibrational frequencies of H adsorbed on different MoS_2_ systems, using finite differences to determine the Hessian matrix. According to the Sabatier principle, a good catalyst should bind a key intermediate in a manner that it is strong enough to allow the reagent H atoms to stay on the MoS_2_ system for reaction but weak enough to enable the release of any produced H_2_ molecules.

### Phase-field simulations: simulation of the drive mechanism: MoS_2_ pushing Au QDs long the growth direction

The equilibrium status of the Au QDs at atomic steps dictates they can be pushed by the MoS_2_ growth front or not, which is crucial to dynamic evolution process. We undertook a case study of the equilibrium status of a Au QD at the edge of a MoS_2_ grain.

We adopt a phase-field model^63^ to simulate how the wetting process influences the equilibrium shape profile. The free energy function is given as,

3 $$\begin{array}{l}F(\rho ) = {\int}_V {[\frac{c}{2}\rho ^2(\rho - 1)^2 + \frac{\kappa }{2}(\nabla \rho )^2 + \lambda \xi (\frac{{\rho ^3}}{3} - \frac{{\rho ^2}}{2})]{\mathrm{d}}V} \end{array}$$

where *ρ* is the phase field for the density of the liquid, *V* is the space volume, *C* = 10 and *κ* = 1 are constants of bulk energy and surface tension, respectively. *ρ* = 1 denotes the liquid phase and *ρ* = 0 denotes the vapor phase. The term $\lambda \xi (\frac{{\rho ^3}}{3} - \frac{{\rho ^2}}{2})$ provides the driving force to the Au particle evolution with a coupling constant *λ* = 180, the dimensionless supersaturation *ξ* is set as (*v*_0_–*v*_t_)/*v*_vapor_, where *v*_0_ is the initial volume of the particle, *v*_t_ is the real time volume of the particle, and *v*_vapor_ is the volume of vapor. The evolution equation in the Allen–Cahn form is,

4 $$\frac{{{\mathrm{d}}\rho }}{{{\mathrm{d}}t}} = \kappa \Delta \rho - C\rho (\rho - 1)(2\rho - 1) + \lambda \xi (\rho - \rho ^2)$$

This evolution equation differs from that of Borcia et al.’s work^63^, which considers full fluid dynamics by including extra terms from the Navier–Stokes equation. In our study, we focus on the equilibrium and do not need to consider the exact evolution kinetics, thus leading to a simpler form of the evolution equation.

We control the contact angle between the Au QD and the substrate by fixing *ρ* = *ρ*_S_ at the substrate. Then, the contact angle can be analytically obtained via the equation $\cos \theta = - 1 + 6\rho _{\mathrm{S}}^2 - 4\rho _{\mathrm{S}}^3$. We set *ρ*_S_ = 0.5 for the MoS_2_ substrate as corresponding to a contact angle between the Au and the substrate of 90°. Likewise, we set *ρ*_S_ = 0.21 for the MoS_2_ substrate as corresponding to a contact angle between the Au and the substrate of 138°.

We solve the evolution equation in Matlab by discretization with a time step of 0.01 and let the simulation run for a long enough time to ensure the system reaches equilibrium. The particle has a diameter of ~11 nm if resting on the MoS_2_ sheet.

The result shows that this Au QD will stay on the lower part of the MoS_2_ atomic step, but keeps in contact with the step edge. This suggests that if in the liquid phase, the Au QD would be pushed forward by the growth front of the second MoS_2_ layer during the growth.

### Phase-field simulations: simulation of the zipper effect suturing the neighbor nanograins

If the Au QDs can be pushed by the growing MoS_2_ grains, they are likely to coalesce when two growth fronts meet, resulting in a complicated evolution. To obtain insights into this process, we perform another phase-field simulation. The phase-field model for the grain evolution and the set of parameters is almost identical to that reported in the literature for a similar system^64^. We ignore the evolution of the supersaturation and keep it at a constant of 0.1 everywhere. A detailed formulation is omitted here for brevity and can be found in other work^65,66^. In addition to the grains, we pay attention to the evolutions of the Au QDs. These QDs are modeled as hemispherical balls sliding on the substrate. For simplicity, we ignore the effect of the Au QDs on the growth of MoS_2_ grains. Then these QDs move on the substrate at a speed of *v* = 50 tan^−1^(*t*)/*π*, where *t* is the dimensionless time of evolution. When two QDs meet, they merge and form a larger QD. The volume of the new QD is the sum of the volumes of the previous two QDs and its center is at the midpoint between the centers of the previous two QDs. We then solve the evolution equation in Matlab by discretization with a time step of 0.01. Initially Au QDs are randomly placed on the substrate and there is in the vicinity of these QDs, a circular nucleus of MoS_2_ with a radius of *r*_0_ and a randomly assigned grain orientation.

To make the simulation consistent with the experiment, we extract some parameters from the experimental data. The experiment shows that the QDs are typically 4–5 nm. It also shows that when observed on top of the MoS_2_ sheet, the Au QDs are larger and less dense by around 2.5 times. This suggests by pushing Au QDs on top of MoS_2_ sheet, the final number of QDs will be 2/5 of the initial QDs. A subtle issue involved in the simulation is the ratio between *n*_Au_ (initial number of Au QDs) and *n*_n_ (the number of nuclei). To address this issue, we take a simulation case study to find a reasonable ratio. By fixing *n*_n_ = 5, we obtain that when *n*_Au_ = 9.2, the final number of QDs is 2/5 of the initial QD number, consistent with the experiments. We therefore choose *n*_Au_ = 10 in further simulations.

### Device fabrication procedure

First, large scale and high-quality single-layer graphene films were grown on Cu foils by CVD, and then transferred onto the prepatterned chips through a standard PMMA-assisted transfer method. Second, electron beam lithography (EBL) and O_2_ plasma were employed to pattern the graphene film into isolated strips with desired size, shape, and location. Third, as-grown MoS_2_ films were transferred on the patterned graphene strips, also using a PMMA-assisted transfer method. For the nanograin films, an additional step carefully positioning an EBL-defined PMMA template was needed. Fourth, an annealing process at 200 °C under high vacuum condition (1 × 10^−5^ Torr) was conducted to remove trapped residual molecules between the graphene and MoS_2_ films to optimize their interfaces. Fifth, EBL followed by thermal evaporation was employed to fabricate the electrodes (Cr/Au, 2 nm/60 nm) on graphene to connect the device with the Au contact pads. Finally, the entire device as fabricated on the chip was passivated by a 500 nm PMMA film, followed by EBL to remove the PMMA above the region of interest in the MoS_2_ film, and expose this section and this section only of the active catalyst to the electrolyte in HER test. Any electrochemical activity only occurs within the exposed window on the nanosheet, while the rest of regions, including the electrodes and the contact pads, are fully passivated by the PMMA to ensure a full electrochemical inertness.

A mirror GB in CVD-grown MoS_2_ is chosen as representative of single GBs, due to the ease with which it can be distinguished in optical images. It also consists mainly of 8|4|4 or defect 8|4|4 rings^3,11^ and has therefore a similar atomic structure to many of the complex boundaries observed in the nanograin films. The length–width ratio of the exposed window for the single-GB device or for the edge device (exposing the edge of an otherwise single-crystalline patch of CVD-grown MoS_2_) is fixed at a ratio of about 2:1, and the length of the window is comparable to the length of the edge or GB in experiments.

### Four-electrode micro-electrochemical measurements

A micro-electrochemical cell with four electrodes (the circuit diagram for this cell is introduced in Supplementary Fig. 20) was developed in our experiment. Among the four electrodes, two serve as counter and reference electrode, using pencil graphite and a Ag/AgCl micro reference electrode (Harvard Apparatus), respectively. The remaining two electrodes were connected to the graphene supporting layer to monitor the conductance signal of graphene and the electrocatalytic signal of MoS_2_ during HER. The device fabrication procedure is shown in Supplementary Figs. 21 and 22, and Supplementary Notes 2 and 3. In all experiments, only the exposed region of the MoS_2_ nanosheets contributes to the recorded HER performance.

The typical four-electrode micro-electrochemical measurements were conducted in a 0.5 M H_2_SO_4_ electrolyte solution. The scan rate is 5 mV step^−1^. Both electrocatalytic current (*I*_c_) and conductance current (*I*_ds_) are simultaneously detected. The leakage current in *I*_c_ is about 10^−10^ A (the electrochemical signal without any exposed window, i.e., from a region with PMMA passivation). The electrochemical current density is calculated by normalizing the current to the area of the exposed window on the MoS_2_. In 0.5 M H_2_SO_4_ solution, we measured:

5 $$E_{{\mathrm{RHE}}} = E_{{\mathrm{Ag}}/{\mathrm{AgCl}}} + 0.219\,{\mathrm{V}}$$

### Standard macro-electrochemical measurements

The macro-electrochemical measurements were conducted on a glassy carbon electrode (3 mm in diameter). Using a similar PMMA-assisted transfer method as described above, we transferred CVD-grown graphene and MoS_2_ nanograin films layer-by-layer on a glassy carbon electrode, and the sample area exceeding to glassy carbon electrode was scraped off before measuments. A standard three electrode system was used, and a Pt plate and Ag/AgCl rod served as counter and reference electrode, respectively. The measurement was performed on a biological electrochemical station in H_2_-saturated 0.5 M H_2_SO_4_ solution. Linear sweep voltammetry (LSV) was conducted at a scan rate of 5 mV s^−1^. The onset potential is defined as the beginning potential of Tafel linear region. The stability test was carried out by taking continuous potential cycling in the potential window of −0.181 to ~0.219 V (versus RHE) at a scan rate of 100 mV s^−1^. The presented LSV curves in macro-electrochemical cell were iR-corrected by subtracting the ohmic resistance loss (about 9 Ω), the value of which is obtained from electrochemical impedance spectroscopy measurement (Supplementary Fig. 32). To evaluate the electrochemically active surface area of the catalysts, the Cu underpotential deposition method^67–70^ was applied, as shown in Supplementary Fig. 30 and Supplementary Table 3.

To test the faradaic efficiency, the H_2_ products were analyzed with an online gas chromatography (GC) setup (Agilent 7890B) equipped with a thermal conductivity detector. Argon (≥99.999%) was used as the carrier gas. The Faradaic efficiency (FE) was calculated by comparing the measured amount of H_2_ generated by cathodal electrolysis with the calculated amount of H_2_ (assuming an FE of 100%), and the equation is given by:

6 $${\mathrm{FE}}\left( {\mathrm{\% }} \right) = \frac{{96485 \times 2 \times {\mathrm{mol}}\,{\mathrm{H}}_2({\mathrm{GC}}) \times 100}}{{\mathrm{Q}}}$$

where the moles of H_2_ is measured by GC (the calibration is needed in advance through injecting high-purity H_2_ to GC, and the H_2_ volume is linearly dependent on GC peak area), Q is obtained from the electrochemical measurements.

### Material characterization

The microstructures and morphologies based on MoS_2_ were characterized by optical microscopy, SEM (FEI 4200), and Raman spectroscopy (Renishaw inVia). Raman spectroscopy was performed with a 514.5 nm laser (with spot size about 1 μm in diameter) at room temperature. The micro-electrochemical measurements were performed using two source meters (Keithley 2400 and 2450). STEM measurements were performed at the SuperSTEM Laboratory, Daresbury, UK, on a Nion UltraSTEM100 aberration-corrected dedicated STEM. The Nion UltraSTEM has a cold field emission gun with a native energy spread of 0.35 eV and was operated at 60 kV acceleration voltage. The beam was set up to a convergence semiangle of 30 mrad with an estimated beam current of ~100 pA. Under these operating conditions, the estimated probe size is ~1.1 Å, providing the perfect tool for atom-by-atom chemical analysis. HAADF imaging was employed to produce atomically resolved images whose intensity is approximately proportional to the square of the average atomic number Z of the material under investigation. This chemically sensitive “Z-contrast” mode is ideally suited to directly identify the nature of individual atoms. All the HAADF images presented here were filtered for visual clarity using a wiener filter with coefficient sigma ranging from 2–5 and 50 iterations in MEM (Maximum Entropy Methods) with fourth order Gaussian filter, as implemented in the STEM-CELL software^71,72^. Geometrical Phase Analysis (GPA) was carried out using a cosine mask of suitable size and 1 binning base. HRTEM and low-resolution annular dark field (ADF)-STEM images were obtained on a FEI Tecnai F20 field emission gun microscope with a 0.19 nm point-to-point resolution at 200 kV, equipped with an embedded Quantum Gatan Image Filter for EELS analyses. Some STEM characterizations were carried out on a JEOL ARM-200F (S)TEM equipped with CEOS CESCOR aberration corrector, operated at an accelerating voltage of 80 kV. The convergence semiangle and acquisition semiangle were 28–33 and 68–280 mrad for the ADF imaging. The dwell time per pixel was set to 12–20.

## Supplementary information

Supplementary Information

Peer Review File

Supplementary Movie 1

Supplementary Movie 2

## Data Availability

The data that support the findings of this study are available from the corresponding author upon reasonable request.

## References

[1] Yu Q (2011). Control and characterization of individual grains and grain boundaries in graphene grown by chemical vapour deposition. Nat. Mater..

[2] Najmaei S (2013). Vapour phase growth and grain boundary structure of molybdenum disulphide atomic layers. Nat. Mater..

[3] van der Zande AM (2013). Grains and grain boundaries in highly crystalline monolayer molybdenum disulphide. Nat. Mater..

[4] Chang S-Y, Chang T-K (2007). Grain size effect on nanomechanical properties and deformation behavior of copper under nanoindentation test. J. Appl. Phys..

[5] Son D-Y (2016). Self-formed grain boundary healing layer for highly efficient CH_3_ NH_3_ PbI_3_ perovskite solar cells. Nat. Energy.

[6] Li C (2014). Grain-boundary-enhanced carrier collection in CdTe solar cells. Phys. Rev. Lett..

[7] Bowman WJ, Zhu J, Sharma R, Crozier PA (2015). Electrical conductivity and grain boundary composition of Gd-doped and Gd/Pr co-doped ceria. Solid State Ion..

[8] Guo CF, Sun T, Liu Q, Suo Z, Ren Z (2014). Highly stretchable and transparent nanomesh electrodes made by grain boundary lithography. Nat. Commun..

[9] Sangwan VK (2015). Gate-tunable memristive phenomena mediated by grain boundaries in single-layer MoS_2_. Nat. Nanotechnol..

[10] Feng X, Jiang K, Fan S, Kanan MW (2015). Grain-boundary-dependent CO_2_ electroreduction activity. J. Am. Chem. Soc..

[11] Li G (2016). All the catalytic active sites of MoS_2_ for hydrogen evolution. J. Am. Chem. Soc..

[12] Seh, Z. W. et al. Combining theory and experiment in electrocatalysis: insights into materials design. *Science* **355**, eaad4998 (2017). 10.1126/science.aad4998 28082532

[13] Voiry D, Yang J, Chhowalla M (2016). Recent strategies for improving the catalytic activity of 2D TMD nanosheets toward the hydrogen evolution reaction. Adv. Mater..

[14] Liu Y (2017). Self-optimizing, highly surface-active layered metal dichalcogenide catalysts for hydrogen evolution. Nat. Energy.

[15] Li H (2016). Activating and optimizing MoS_2_ basal planes for hydrogen evolution through the formation of strained sulphur vacancies. Nat. Mater..

[16] Kibsgaard J, Chen Z, Reinecke BN, Jaramillo TF (2012). Engineering the surface structure of MoS_2_ to preferentially expose active edge sites for electrocatalysis. Nat. Mater..

[17] Ji Q, Zhang Y, Zhang Y, Liu Z (2015). Chemical vapour deposition of group-VIB metal dichalcogenide monolayers: engineered substrates from amorphous to single crystalline. Chem. Soc. Rev..

[18] Jaramillo TF (2007). Identification of active edge sites for electrochemical H_2_ evolution from MoS_2_ nanocatalysts. Science.

[19] Xie J (2013). Defect-rich MoS_2_ ultrathin nanosheets with additional active edge sites for enhanced electrocatalytic hydrogen evolution. Adv. Mater..

[20] Dai X (2015). Co-doped MoS_2_ nanosheets with the dominant CoMoS phase coated on carbon as an excellent electrocatalyst for hydrogen evolution. ACS Appl. Mater. Interfaces.

[21] Voiry D (2016). The role of electronic coupling between substrate and 2D MoS_2_ nanosheets in electrocatalytic production of hydrogen. Nat. Mater..

[22] Ye G (2016). Defects engineered monolayer MoS_2_ for improved hydrogen evolution reaction. Nano Lett..

[23] Ouyang Y (2016). Activating inert basal planes of MoS_2_ for hydrogen evolution reaction through the formation of different intrinsic defects. Chem. Mater..

[24] Li S (2018). Vapour–liquid–solid growth of monolayer MoS_2_ nanoribbons. Nat. Mater..

[25] Zhou J (2018). A library of atomically thin metal chalcogenides. Nature.

[26] Gong Y (2016). Synthesis of millimeter-scale transition metal dichalcogenides single crystals. Adv. Funct. Mater..

[27] He Y (2016). Layer engineering of 2D semiconductor junctions. Adv. Mater..

[28] Wang X (2014). Chemical vapor deposition growth of crystalline monolayer MoSe_2_. ACS Nano.

[29] Gao Y (2017). Ultrafast growth of high-quality monolayer WSe_2_ on Au. Adv. Mater..

[30] Li S (2015). Halide-assisted atmospheric pressure growth of large WSe_2_ and WS_2_ monolayer crystals. Appl. Mater. Today.

[31] Dathbun A (2017). Large-area CVD-grown sub-2 V ReS_2_ transistors and logic gates. Nano Lett..

[32] Zhou J (2017). Large-area and high-quality 2D transition metal telluride. Adv. Mater..

[33] Naylor CH (2016). Monolayer single-crystal 1T’-MoTe_2_ grown by chemical vapor deposition exhibits weak antilocalization effect. Nano Lett..

[34] Cui F (2017). Epitaxial growth of large-area and highly crystalline anisotropic ReSe_2_ atomic layer. Nano Res..

[35] Gao Y (2015). Large-area synthesis of high-quality and uniform monolayer WS_2_ on reusable Au foils. Nat. Commun..

[36] Empante TA (2017). Chemical vapor deposition growth of few-layer MoTe_2_ in the 2H, 1T’, and 1T phases: tunable properties of MoTe_2_ films. ACS Nano.

[37] Feng Q (2014). Growth of large-area 2D MoS_2(1-x)_Se_2x_ semiconductor alloys. Adv. Mater..

[38] Wu S (2013). Vapor–solid growth of high optical quality MoS_2_ monolayers with near-unity valley polarization. ACS Nano.

[39] Huang J-H (2017). Large-area 2D layered MoTe_2_ by physical vapor deposition and solid-phase crystallization in a tellurium-free atmosphere. Adv. Mater. Interfaces.

[40] Kang K (2015). High-mobility three-atom-thick semiconducting films with wafer-scale homogeneity. Nature.

[41] Kang K (2017). Layer-by-layer assembly of two-dimensional materials into wafer-scale heterostructures. Nature.

[42] Lee S-H, Kwak E-H, Jeong G-H (2015). Dewetting behavior of electron-beam-deposited Au thin films on various substrates: graphenes, quartz, and SiO_2_ wafers. Appl. Phys. A.

[43] Seguini G (2014). Solid-state dewetting of ultra-thin Au films on SiO_2_ and HfO_2_. Nanotechnology.

[44] Li H (2012). From bulk to monolayer MoS_2_: evolution of Raman scattering. Adv. Funct. Mater..

[45] Huang PY (2011). Grains and grain boundaries in single-layer graphene atomic patchwork quilts. Nature.

[46] Shi J, Ji Q, Liu Z, Zhang Y (2016). Recent advances in controlling syntheses and energy related applications of MX_2_ and MX_2_/graphene heterostructures. Adv. Energy Mater..

[47] Shi Y, Li H, Li L-J (2015). Recent advances in controlled synthesis of two-dimensional transition metal dichalcogenides via vapour deposition techniques. Chem. Soc. Rev..

[48] Ruffino F, Grimaldi MG (2015). Controlled dewetting as fabrication and patterning strategy for metal nanostructures. Phys. Status Solidi A.

[49] Choi, H.-J. *Vapor–Liquid–Solid Growth of Semiconductor Nanowires* (Springer Berlin Heidelberg, 2012).

[50] Kresse G, Hafner J (1994). Ab initio molecular-dynamics simulation of the liquid-metal-amorphous-semiconductor transition in germanium. Phys. Rev. B.

[51] Lauritsen JV (2007). Size-dependent structure of MoS_2_ nanocrystals. Nat. Nanotechnol..

[52] Nørskov JK (2005). Trends in the exchange current for hydrogen evolution. J. Electrochem. Soc..

[53] Zhou W (2013). Intrinsic structural defects in monolayer molybdenum disulfide. Nano Lett..

[54] Hansen LP (2011). Atomic-scale edge structures on industrial-style MoS_2_ nanocatalysts. Angew. Chem. Int. Ed..

[55] Yu WJ (2013). Vertically stacked multi-heterostructures of layered materials for logic transistors and complementary inverters. Nat. Mater..

[56] Allain A, Kang J, Banerjee K, Kis A (2015). Electrical contacts to two-dimensional semiconductors. Nat. Mater..

[57] Kang J, Liu W, Sarkar D, Jena D, Banerjee K (2014). Computational study of metal contacts to monolayer transition-metal dichalcogenide semiconductors. Phys. Rev. X.

[58] Wang J (2017). Field effect enhanced hydrogen evolution reaction of MoS_2_ nanosheets. Adv. Mater..

[59] Ding M (2015). An on-chip electrical transport spectroscopy approach for *in situ* monitoring electrochemical interfaces. Nat. Commun..

[60] He Y (2019). Self-gating in semiconductor electrocatalysis. Nat. Mater..

[61] Voiry D (2013). Conducting MoS_2_ nanosheets as catalysts for hydrogen evolution reaction. Nano Lett..

[62] Nsimama PD, Herz A, Wang D, Schaaf P (2016). Influence of the substrate on the morphological evolution of gold thin films during solid-state dewetting. Appl. Surf. Sci..

[63] Borciaa R, B. ID, Bestehorn M (2009). Static and dynamic contact angles- a phase field modelling. Eur. Phys. J. Spec. Top..

[64] Artyukhov VI, Hu Z, Zhang Z, Yakobson BI (2016). Topochemistry of Bowtie- and star-shaped metal dichalcogenide nanoisland formation. Nano Lett..

[65] Steinbach I, Pezzolla F (1999). A generalized field method for multiphase transformations using interface fields. Phys. D Nonlinear Phenom..

[66] Ingo S (2009). Phase-field models in materials science. Modell. Simul. Mater. Sci. Eng..

[67] Zheng Y (2016). High electrocatalytic hydrogen evolution activity of an anomalous ruthenium catalyst. J. Am. Chem. Soc..

[68] Mahmood J (2017). An efficient and pH-universal ruthenium-based catalyst for the hydrogen evolution reaction. Nat. Nanotechnol..

[69] Green CL, Kucernak A (2002). Determination of the platinum and ruthenium surface areas in platinum−ruthenium alloy electrocatalysts by underpotential deposition of copper. I. unsupported catalysts. J. Phys. Chem. B.

[70] Colmenares L, Jusys Z, Behm RJ (2006). Electrochemical surface characterization and O_2_ reduction kinetics of Se surface-modified Ru nanoparticle-based RuSe_y_/C catalysts. Langmuir.

[71] Grillo V, Rotunno E (2013). STEM-CELL: A software tool for electron microscopy: Part I—simulations. Ultramicroscopy.

[72] Grillo V, Rossi F (2013). STEM-CELL: A software tool for electron microscopy. Part 2 analysis of crystalline materials. Ultramicroscopy.

[73] Zhan Y, Liu Z, Najmaei S, Ajayan PM, Lou J (2012). Large-area vapor-phase growth and characterization of MoS_2_ atomic layers on a SiO_2_ substrate. Small.

[74] Kong D (2013). Synthesis of MoS_2_ and MoSe_2_ films with vertically aligned layers. Nano Lett..

[75] Lin Y-C (2014). Direct synthesis of van der Waals solids. ACS Nano.

[76] Song J-G (2013). Layer-controlled, wafer-scale, and conformal synthesis of tungsten disulfide nanosheets using atomic layer deposition. ACS Nano.

[77] Yim C (2016). High-performance hybrid electronic devices from layered PtSe_2_ films grown at low temperature. ACS Nano.

[78] Liu K-K (2012). Growth of large-area and highly crystalline MoS_2_ thin layers on insulating substrates. Nano Lett..

