# Supplementary material for: Engineering grain boundaries at the 2D limit for the hydrogen evolution reaction
Source: Nat Commun. 2020 Jan 2;11:57. doi: 10.1038/s41467-019-13631-2 (PMC6940382; doi:10.1038/s41467-019-13631-2)
Supplement: Supplementary file 1 — Supplementary Information [file 41467_2019_13631_MOESM1_ESM.pdf]

Supplementary Information

Engineering grain boundary at 2D limit for hydrogen evolution reaction

He *et al.*

Supplementary Figures

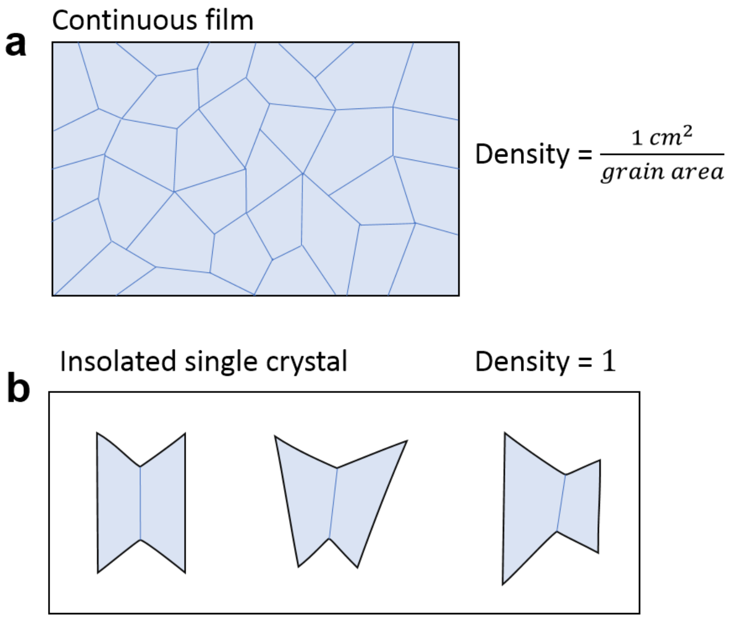

Supplementary Figure 1. Schematic of grain size and density data collected from previously reported TMD continuous film (a) and insolated single crystal (b). In our collection, the data of grain sizes are obtained from optical, SEM, or TEM/STEM images. As for the continuous film, the data of grain density is calculated on grains per unit area ($\frac{1\,cm^2}{grain\ area}$), as shown in a. However, as for the insolated single crystal, their GB's density is usually 1 and is independent of grain size, as shown in b.

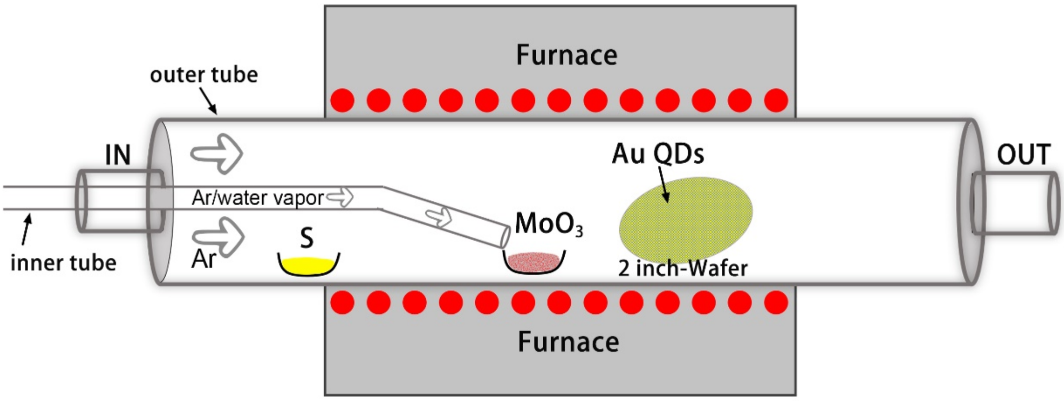

Supplementary Figure 2. Schematic illustration of the home-built CVD system for the growth of water-size MoS_2 films. The use of an inner tube can provide a steady supply of the Mo source but stop the complete sulfurization of the MoO_3 powder through the addition of water vapor to the carrier gas mix aimed at the MoO_3 boat. Downstream from the sources, a high density of Au QDs forms on the wafer-scale support during the temperature ramp period. The formed QDs will facilitate the growth of the MoS_2 nanograin film.

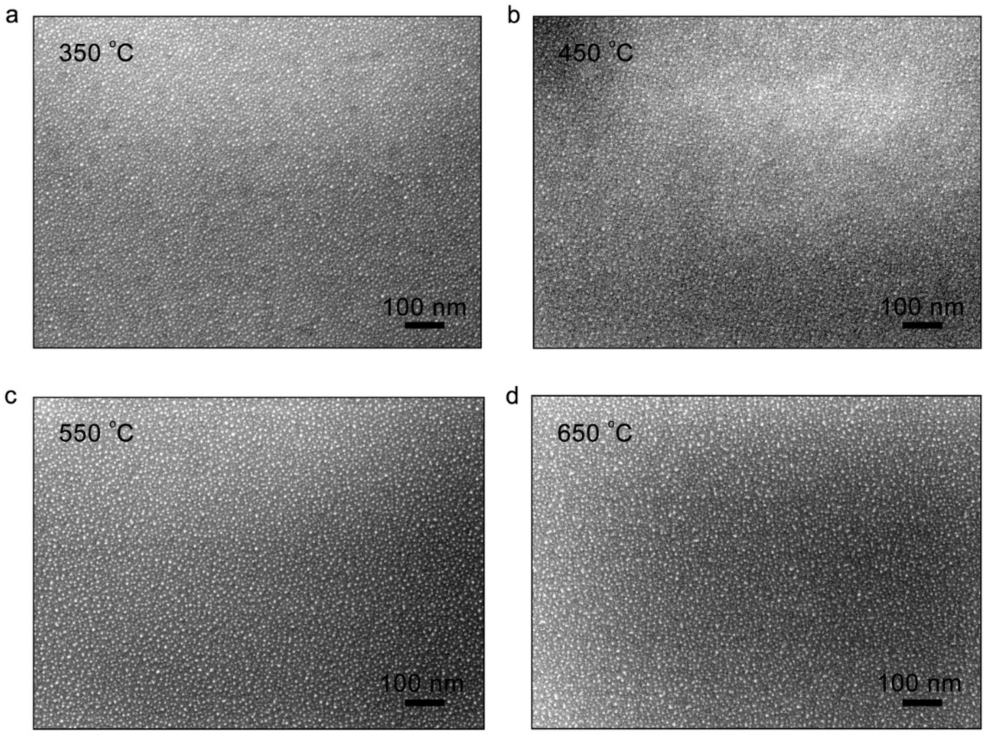

Supplementary Figure 3. The morphology of Au nanoparticles on SiO_2/Si substrate annealed at temperatures of 350 °C (a), 450 °C (b), 550 °C (c), 650 °C (d). In our experiment, a Au film (30 s of deposition time) can agglomerate into Au QDs at a very low temperature (350 °C), as shown in a. No obvious change in the sizes and density of the formed Au QDs is observed when the annealing temperature further increases from 450 °C to 650 °C (b-d). These results suggest that there exists a solid-state dewetting behavior for Au on SiO_2/Si substrate at high temperature. The Au QDs rather than the initial continuous Au film are therefore effectively assisting the growth of MoS_2, since the growth of MoS_2 is carried out at 750 °C in our experiments.

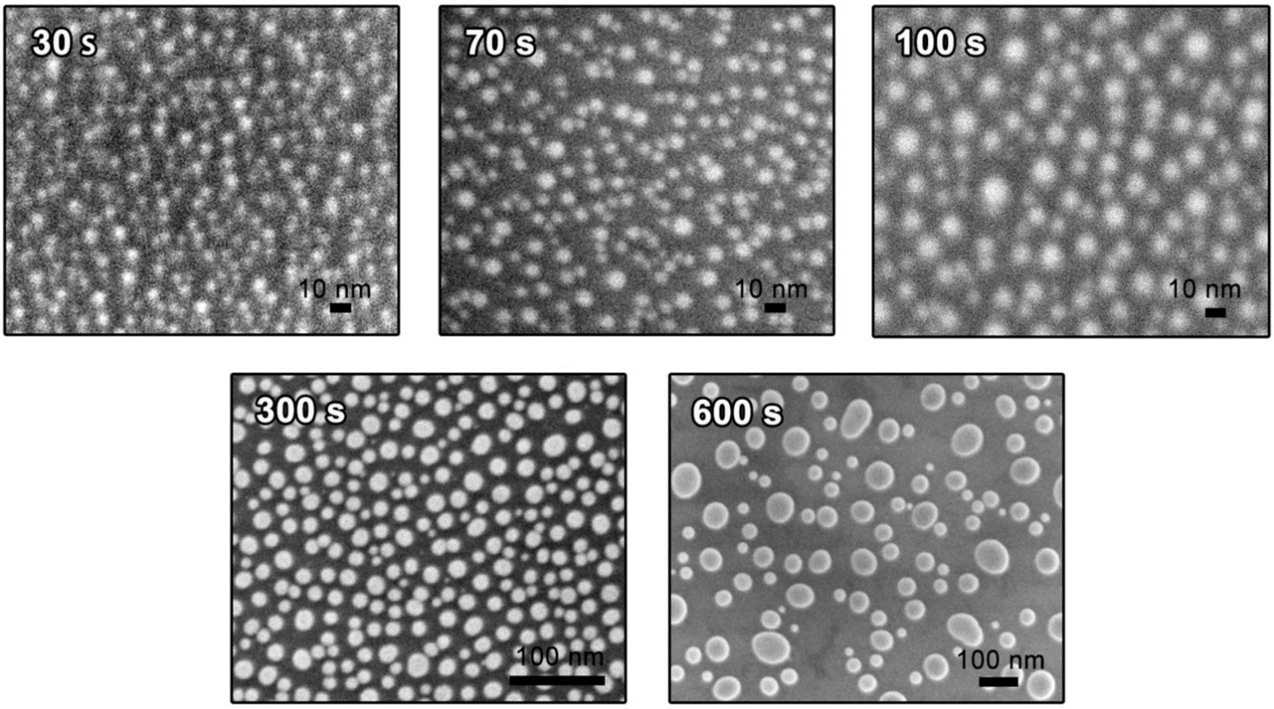

Supplementary Figure 4. SEM images of Au nanoparticles on SiO_2/Si substrate obtained from deposition of Au films over increasing deposition times. By properly adjusting the deposition time of Au by e-beam evaporation with a rate of 0.1 Å s^{-1}, the size and the density of the Au nanoparticles can be finely tuned. For a 30 s deposition time, the obtained QDs exhibit an average diameter of 4.8 nm and a density of 2×10^{12} cm^{-2} (the first image).

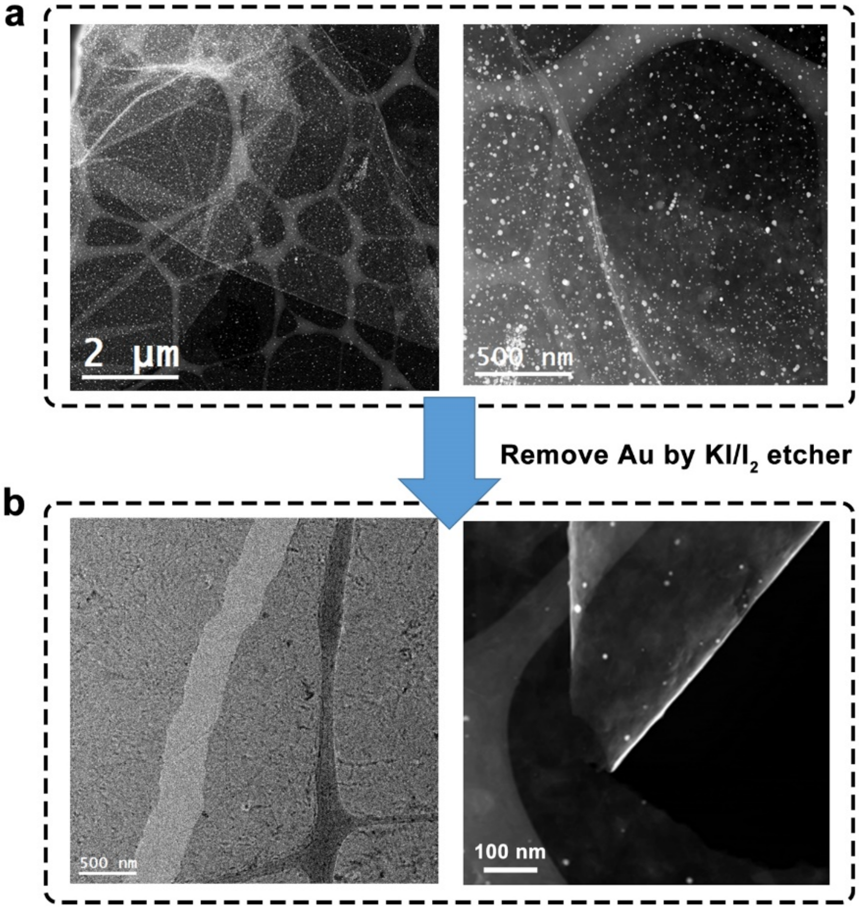

Supplementary Figure 5. TEM images of the MoS_2 film before (a) and after (b) removal of Au. By using a KI/I_2 etch for 40-60 min at room temperature, Au nanoparticles can be efficiently removed from nanograin MoS_2 films.

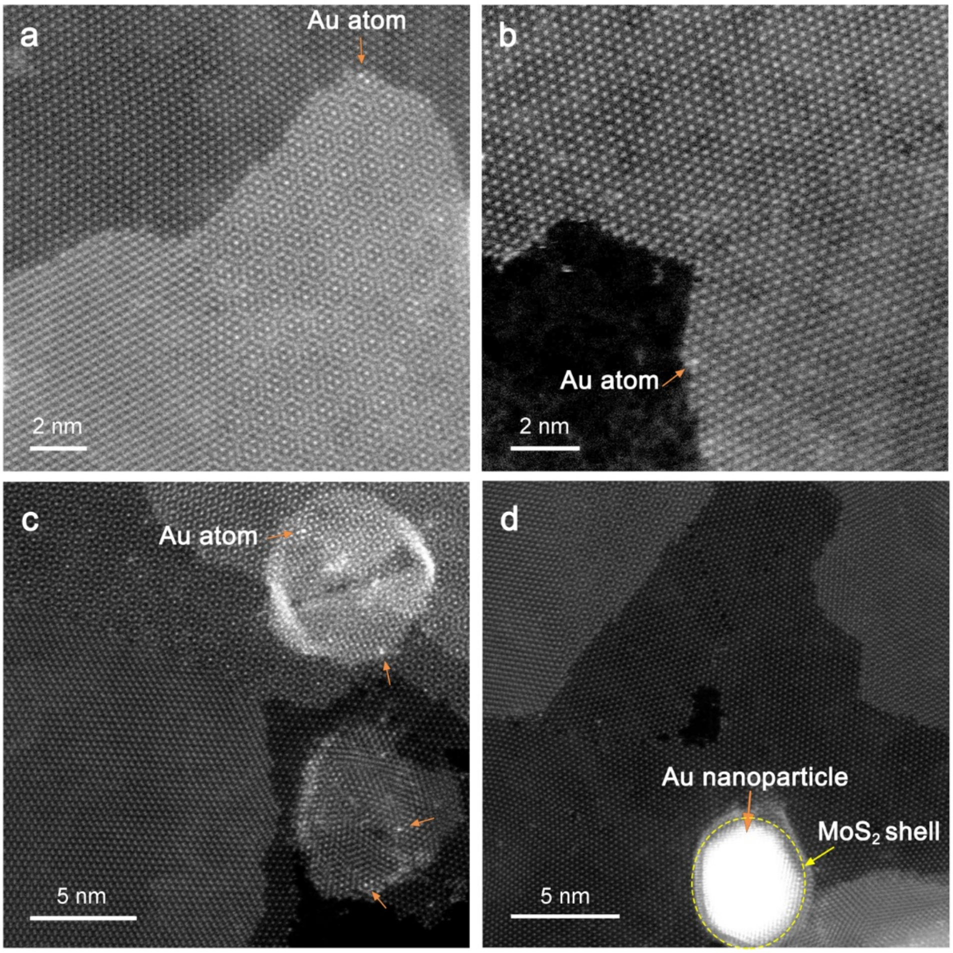

Supplementary Figure 6. Atomically resolved HAADF scanning transmission electron microscopy (STEM) images of the MoS_2 nanograin film after removal of Au. (a-c) A number of much brighter individual atoms observed on the samples (bright spots indicated by arrows on the images) at the edge of grains (a-b) or hollow MoS_2 spheres (c). The small white bright spots correspond to Au atom. The. The MoS_2 hollow spheres are created from the removal of Au nanoparticles. From the STEM images, only a very small number of "likely" Au atoms can be observed, suggesting that most of Au has been etched. (d) Au nanoparticles that are fully coated by MoS_2. A very small amount of residual Au single atoms (white bright spots) (a-c) and nanoparticles (d) can be found in the film. The Au nanoparticles are fully coated by MoS_2. Therefore, it is difficult to remove Au due to the protection of MoS_2. More often, we observed hollow spheres which indicate that the Au nanoparticles inside has been successfully etched. From the STEM images, only a very small amount of Au atoms can be observed, suggesting that most of Au has been etched away.

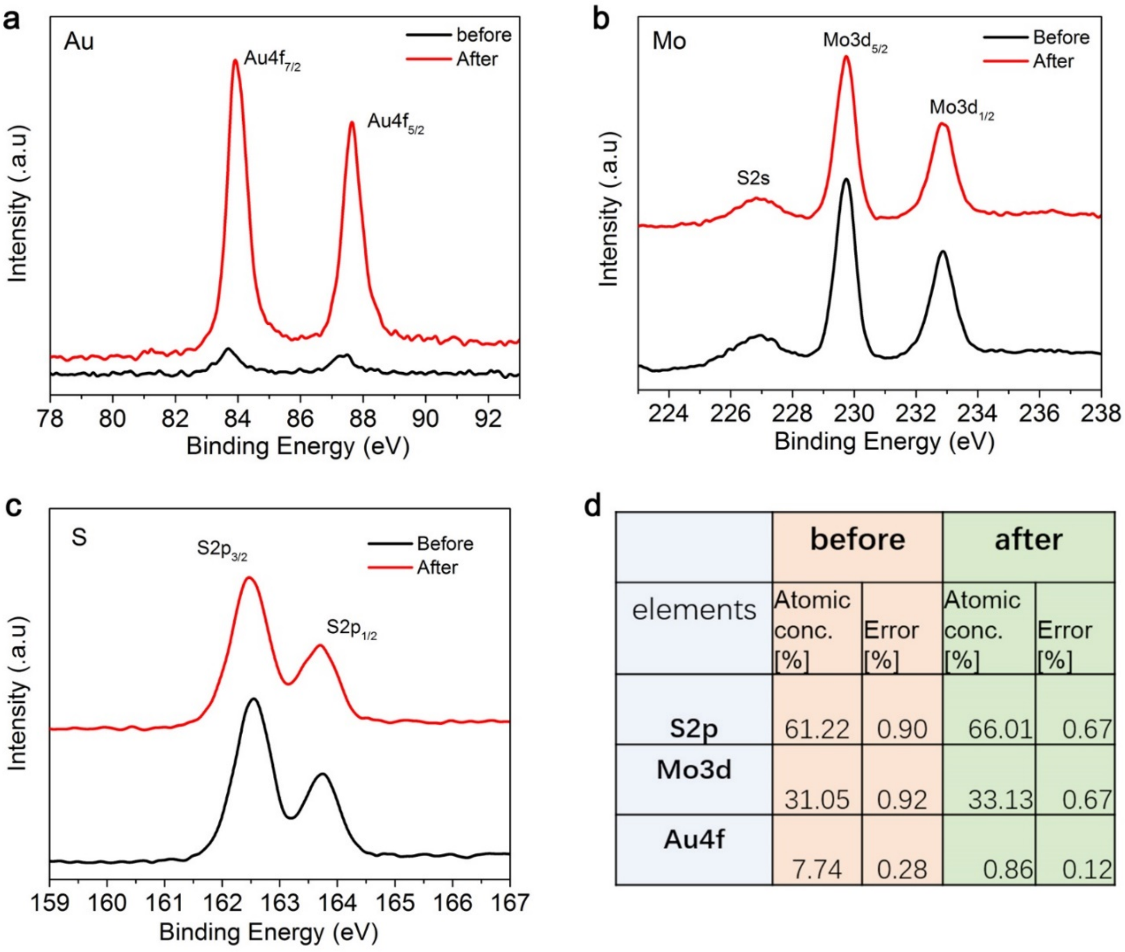

	before		after	
elements	Atomic conc. [%]	Error [%]	Atomic conc. [%]	Error [%]
**S2p**	61.22	0.90	66.01	0.67
**Mo3d**	31.05	0.92	33.13	0.67
**Au4f**	7.74	0.28	0.86	0.12

Supplementary Figure 7. XPS of the MoS_2 nanograin film before and after removal of Au. (a-c) XPS spectra of Au4f, Mo3d, and S2p before and after removing Au by KI/I_2 etcher. (d) Atomic ratio in the MoS_2 nanograin film before and after removal of Au. A 0.86% of Au atomic ratio in the etched film suggests the KI/I_2 etcher can effectively remove the majority of the Au nanoparticles from the as-grown MoS_2 nanograin film.

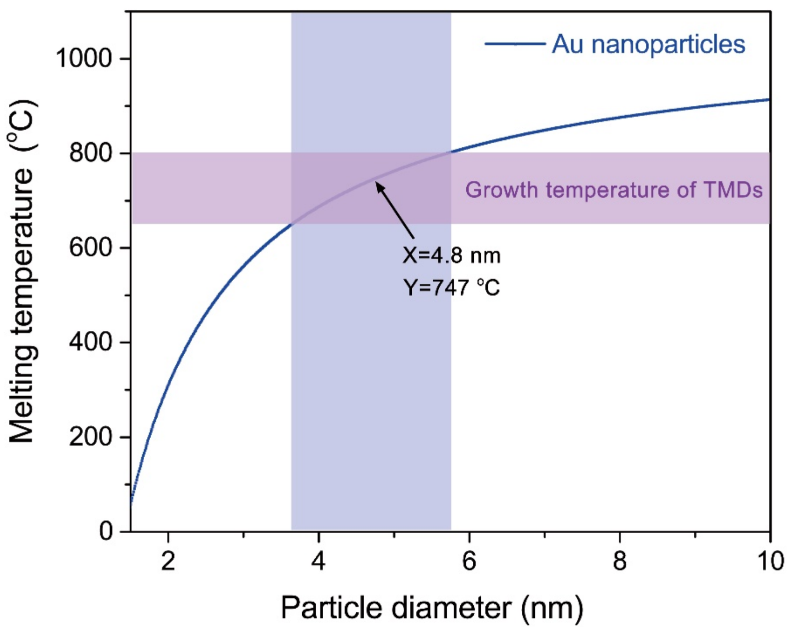

Supplementary Figure 8. The relationship between the diameter and the melting point of Au nanoparticle. According to the literature[1], the melting temperature of a Au metal nanoparticle is given by $T_m = T_{mb}(1-\frac{\beta}{d})$, where β=1.1281 nm for Au, T_{mb}=1337.6 K is the bulk melting temperature of Au, and *d* is the particle's diameter. It can be seen from figure that the melting point of 747 °C for a critical diameter of 5 nm Au nanoparticle can be obtained. Since the growth temperature is about 750-800 °C in our experiments, most of the Au QDs are liquid droplets during the growth process.

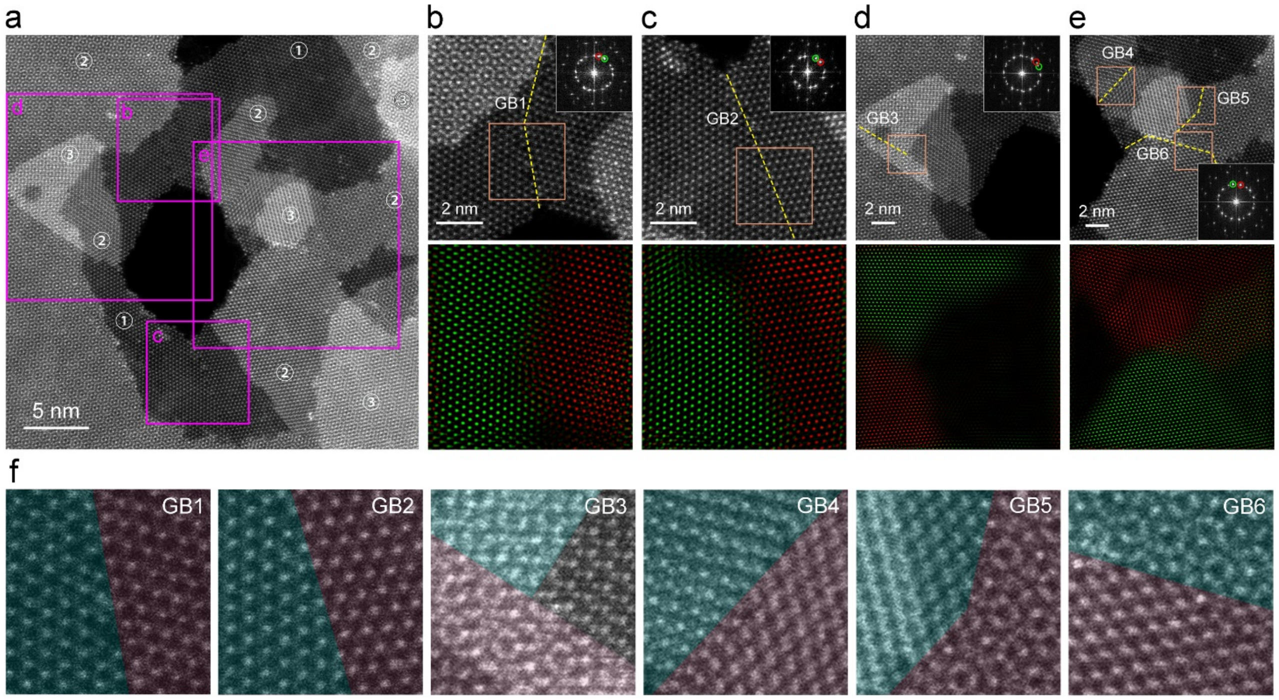

Supplementary Figure 9. STEM investigation of GBs at each layer of few-layer MoS_2 nanograin films. (a) Atomic resolution HAADF STEM image of an area of the nanograin films, exhibiting patches of single, bilayer, and trilayer MoS_2 that were marked as ①, ②, and ③, respectively. (b-e) Top panel: High magnification HAADF STEM images obtained from the pink line squared regions in (a), as indicated. Inset of the top panel: FFT of the corresponding b-e HAADF STEM images. Bottom panel: False-colored frequency-filtered images obtained from the brown line squared regions in (b-e) HAADF STEM images, obtained using the Fourier spots indicated by the color circles on the inset FFTs. The GBs are marked by yellow dash line as a guide to the eye in these STEM images, and exist in 1st, 2nd, or 3rd MoS_2 layer. (f) False-colored images obtained from the brown line squared regions in the **b-e** STEM images.

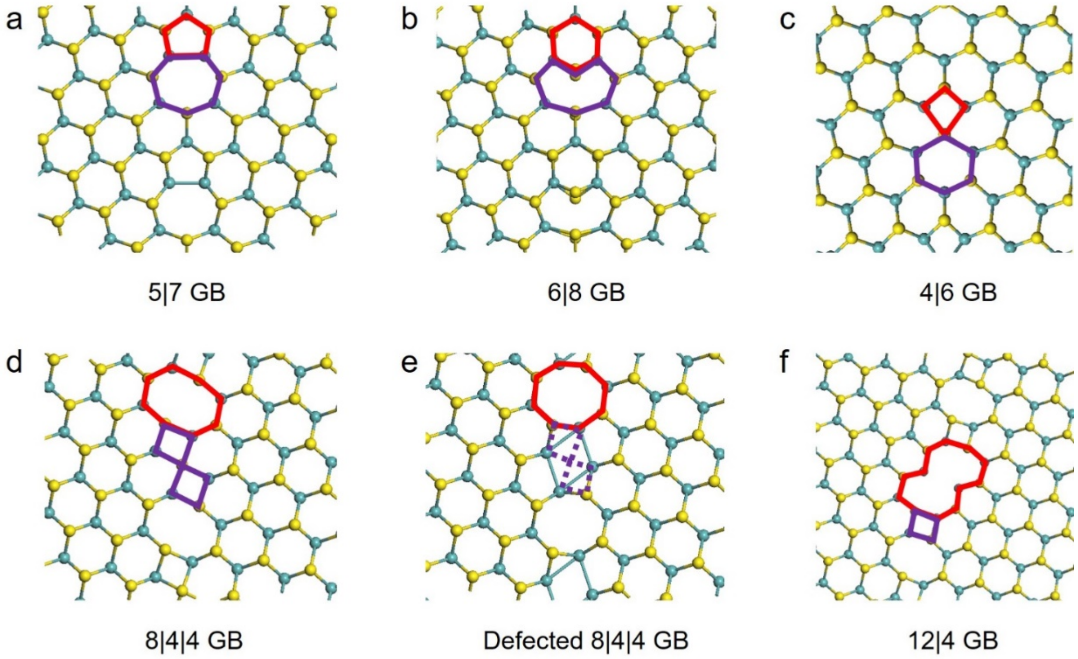

Supplementary Figure 10. Schematic illustration of atomic structures of 5|7 GB, 6|8 GB, 4|6 GB, 8|4|4 GB, Defected 8|4|4 GB, and 12|4 GB in MoS_2. 5|7 GB is formed from 5- and 7- membered rings, which is highlighted by the overlaid red pentagon and purple heptagon in a, with a recurring periodic 5-7 ring motif. 6|8 GB is formed from 6- (red hexagon in b) and 8- (purple octagon in b) membered rings, with a recurring periodic 6-8 ring motif. 4|6 GB is formed from 4- (red tetragon in c) and 6- (purple hexagon in c) membered rings, with a recurring periodic 4-6 ring motif. 8|4|4 GB is formed from 8- (red octagon in d) and two 4- (purple tetragons in d) membered rings, with a recurring periodic 8-4-4 ring motif. Defected 8|4|4 GB is formed by removing two sulphur atoms which connect two 4-membered rings from 8|4|4 GB, as shown in e. 12|4 GB is formed from 12- (red dodecagon in f) and 4- (purple tetragon in f) membered rings.

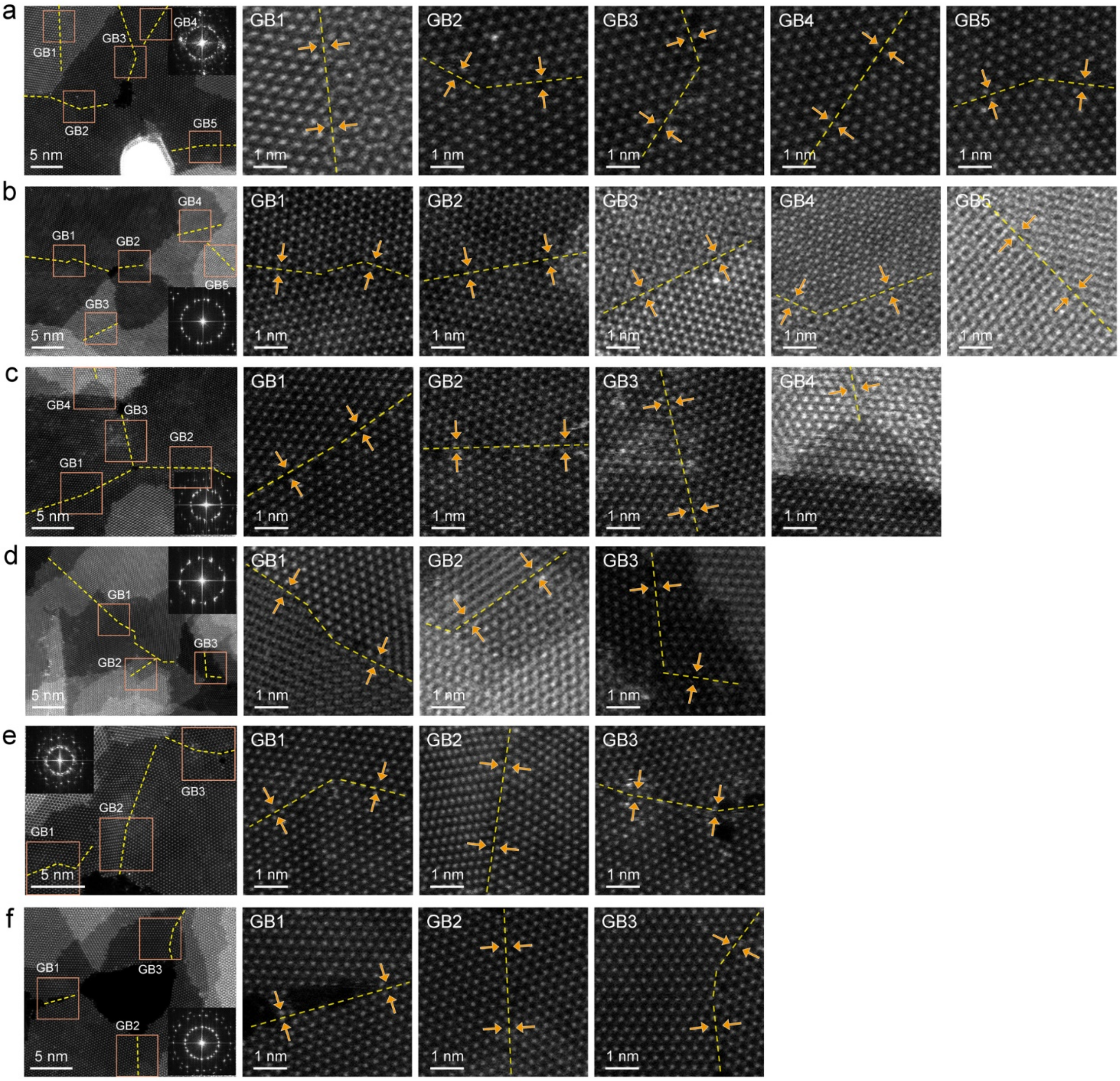

Supplementary Figure 11. STEM investigation of GBs in six different regions of a few-layer MoS_2 nanograin film. The GBs are indicated by pairs of brown arrows in HAADF-STEM images and dashed yellow lines as a guide to the eye. It can be seen that 3 - 5 GBs can be frequently observed within a ~ 400 nm^2 area of the MoS_2 nanograin film.

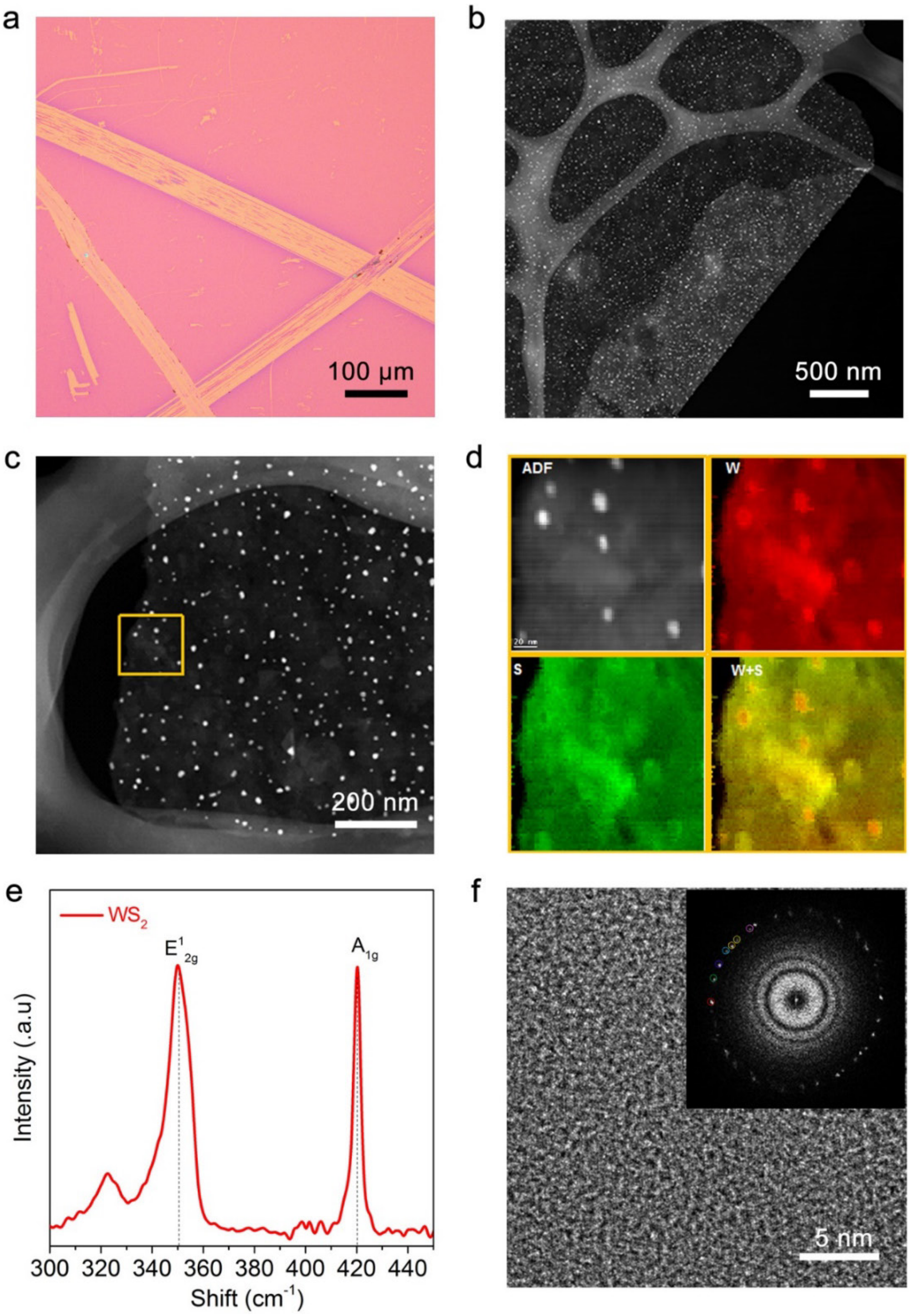

Supplementary Figure 12. WS_2 nanograin film. (a)(b) Optical (a) and ADF STEM (b) images of an as-grown WS_2 nanograin film. (c)(d) EELS chemical composition maps (d) obtained from the yellow rectangular area in (c), showing individual W (Red) and S (Green) maps and their composite. (e) Raman spectrum of the WS_2 nanograin film. (f) HRTEM image and corresponding power spectrum (FFT) of the WS_2 atomic layer, showing it contains 7 different domains or grains rotated by 15.8°, 25.92°, 36.69°, 41.62°, 47.08° and 58.80° within a nearly 600 nm^2 region. This suggests an average sub-10 nm grain size.

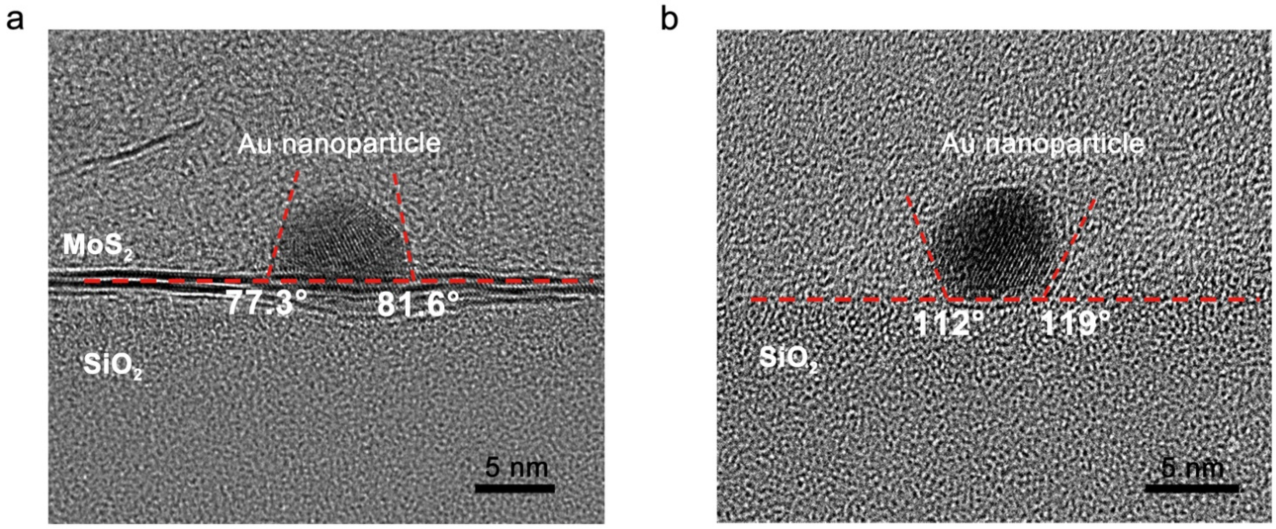

Supplementary Figure 13. Cross-sectional HRTEM images of the Au QD/MoS_2 interface after MoS_2 growth (a) and Au QD/SiO_2 interface prior to the growth (b). The wetting angle of Au on MoS_2 (77.3°-81.6°) is much smaller than that of Au on SiO_2 (112°-119°). This result indicates a much more hydrophobic interface between SiO_2 and Au, leading to a spherical shape of the Au QD.

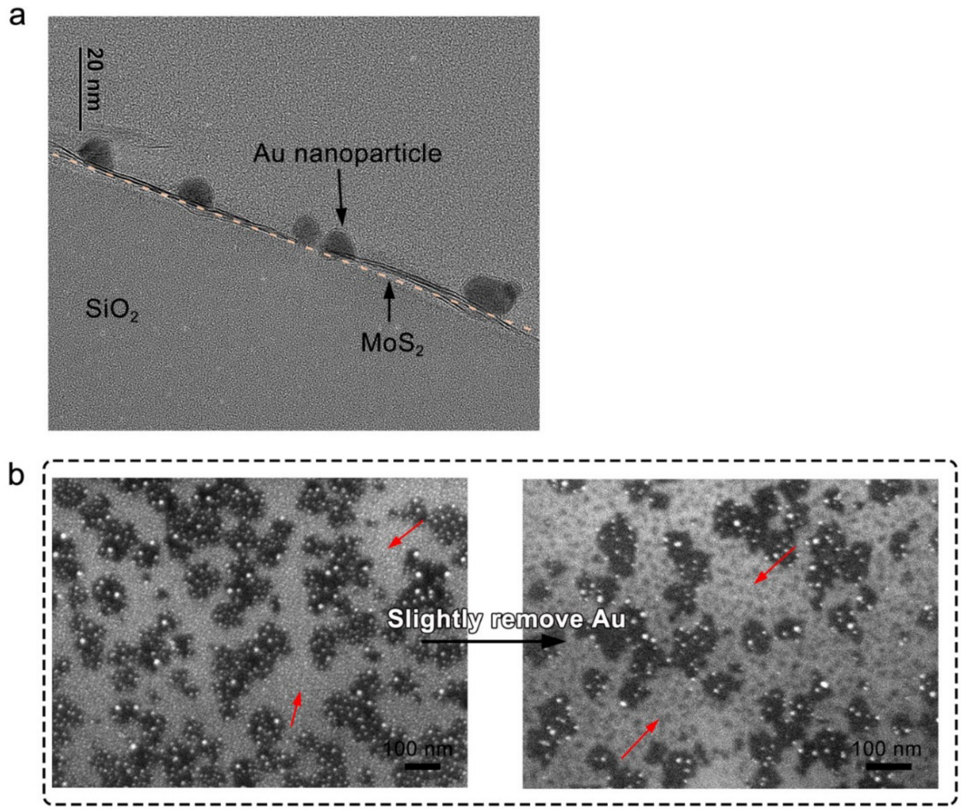

Supplementary Figure 14. Investigation of the relative location between the Au QD and the MoS_2 layer during the growth process. (a) Low-magnification cross-sectional TEM image of as-grown MoS_2 nanograin film, in which all the Au nanoparticles are on the surface of the MoS_2 layer. (b)(c) HRTEM images illustrating the morphology of the as-grown substrate, before (b) and after (c) an incomplete removal of the Au nanoparticles.The phase field simulation suggests that the liquid-phase Au QDs will migrate swiftly from the SiO_2 substrate onto the MoS_2 surface once it encounters the MoS_2 growth front edge during the growth process. This can be experimentally identified through low-magnification cross-section TEM images a), in which all of the Au nanoparticles are on the surface of the MoS_2 layer and no longer in direct contact with the substrate beneath it. This can be also experimentally identified through the footprint of the Au nanoparticle in SEM images (b-c), in which the Au nanoparticles are only partially removed by the etcher (KI/I_2).

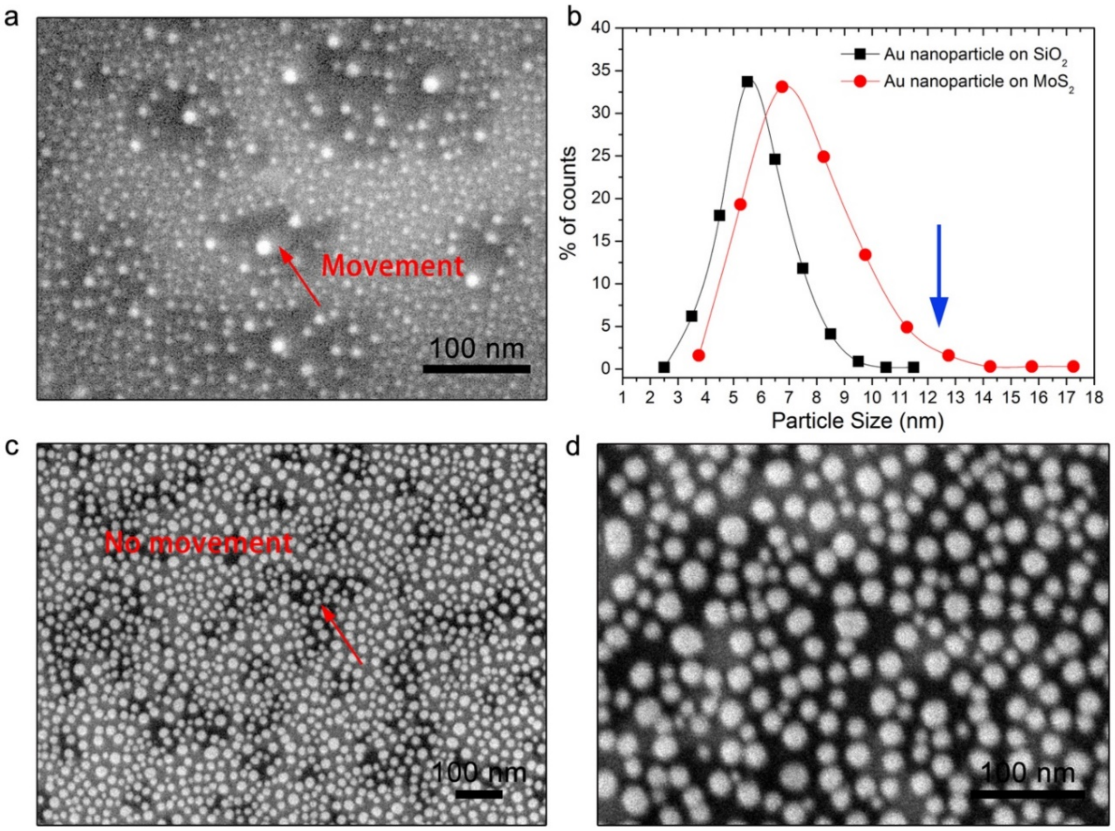

Supplementary Figure 15. Experimental observation of the MoS_2 growth front pushing the Au QDs. (a) SEM images of as-grown MoS_2 from Au QDs. (b) The statistical distribution of the Au nanoparticle size (in diameter) on MoS_2 and SiO_2 in (a). (c)(d) SEM images of as-grown MoS_2 from Au nanoparticles. **a-b** show the size of Au QDs on MoS_2 is usually larger than on SiO_2/Si. Some QDs are clearly seen to be located at the MoS_2 edge. This is because the MoS_2 layer will drive the Au QD droplets along its growth direction; QDs then coalesce into larger Au particles if brought in contact with one another. Therefore, Au QDs will increase in size and decrease in density as the growth of MoS_2 proceeds. It is worth mentioning that we did not observe any obvious change in Au nanoparticle size and density when using different annealing temperatures (Supplementary Figure 3), suggesting that the growth of MoS_2 is the dominant factor in mobilizing the Au QDs. On the other hand, no obvious change in the size or the density of initially larger Au nanoparticles was observed after growth for particles with an average of 15 nm in diameter, as shown in **c-d**. This indicates no movement of these larger Au nanoparticle during MoS_2 growth, which is interpreted as being due to the solid state of Au nanoparticle at the growth temperature in our experiment.

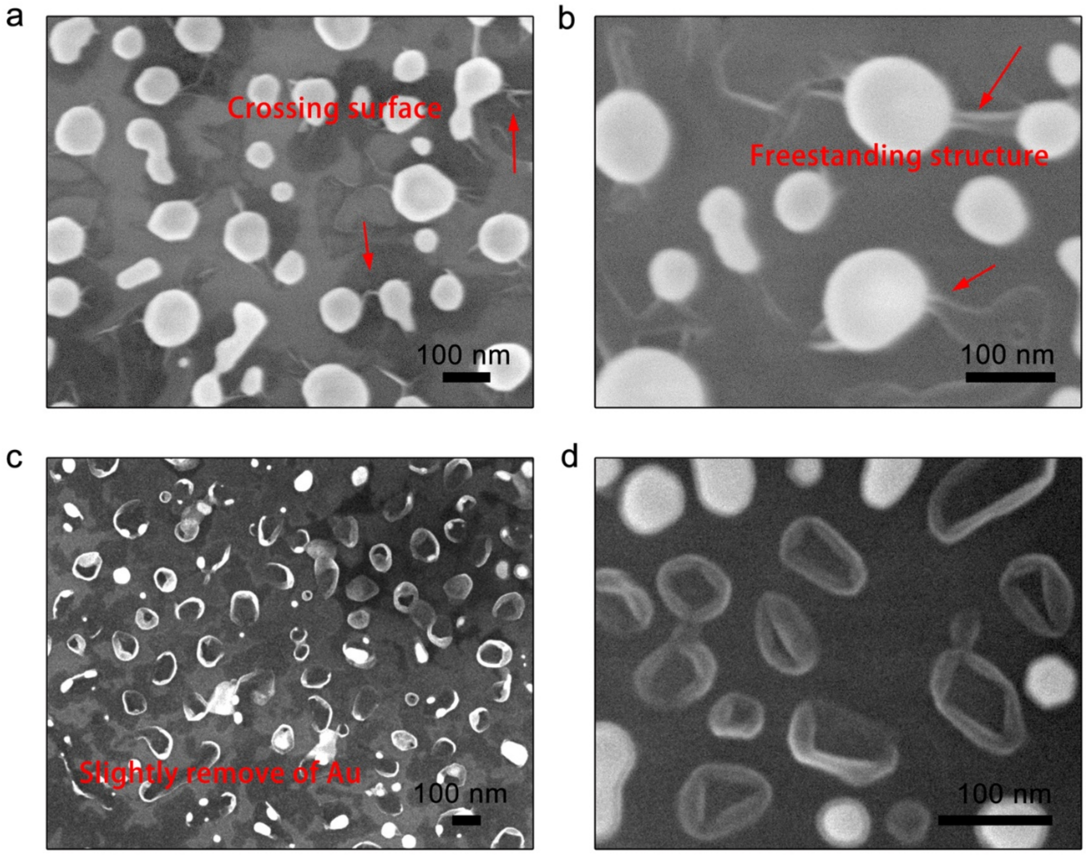

Supplementary Figure 16. The morphology of as-grown MoS_2 before (a-b) and after (c-d) removal of the Au nanoparticles (600 s of deposition time). As shown in a-b, some suspended MoS_2 layers were observed at the edge of large Au nanoparticles (about 100 nm in diameter). c-d show a shrunken ball-like MoS_2 structure left behind after removal of the Au nanoparticles. These observations strongly support the conclusion that MoS_2 grows over the surfaces of large Au nanoparticle but does not drive these particles during the growth process.

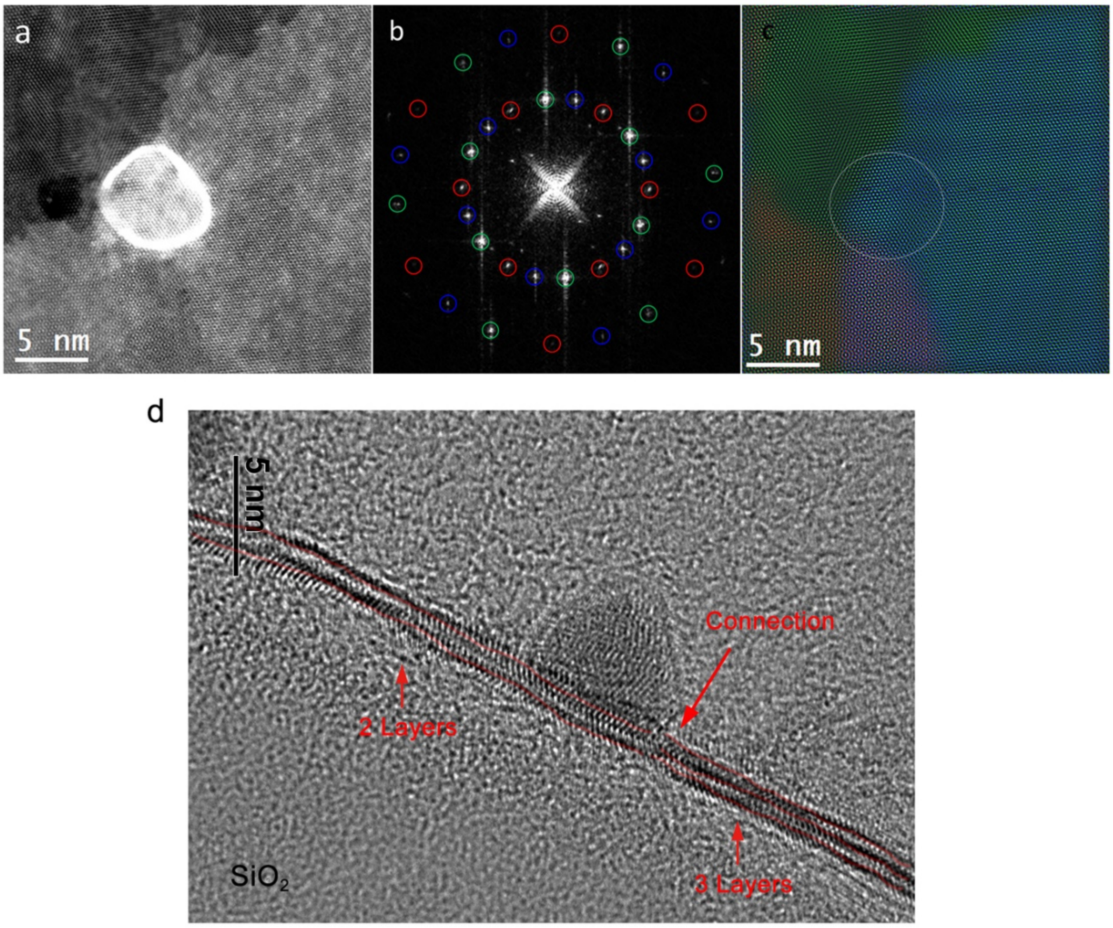

Supplementary Figure 17. Experimental observation of the “zipper” effect. (a) Top-view HAADF-STEM image of an Au nanoparticle/MoS_2 interface. (b) FFT of (a) showing three sets of diffraction spots corresponding to three distinct domains. (c) Composite false-colored frequency filtered image generated by filtering these three sets of diffraction spots marked with a frequency spot mask, revealing the relative positions of the three grains. (d) Cross-sectional HRTEM image of an Au nanoparticle/MoS_2 interface. As shown in **a-c**, Au nanoparticles are often found at the intersection between three MoS_2 grains. A similar configuration can also be seen from the cross-sectional HRTEM image in **d**, where a Au nanoparticle is found at the boundary between a region of the nanograin film that is 2 layers thick (on the left-hand side) and a 3 layer-thick MoS_2 grains. Those results indicate that there exists a zipper effect to suture neighbor nanograins, which is in agreement with the phase field simulations (see Experiment Section).

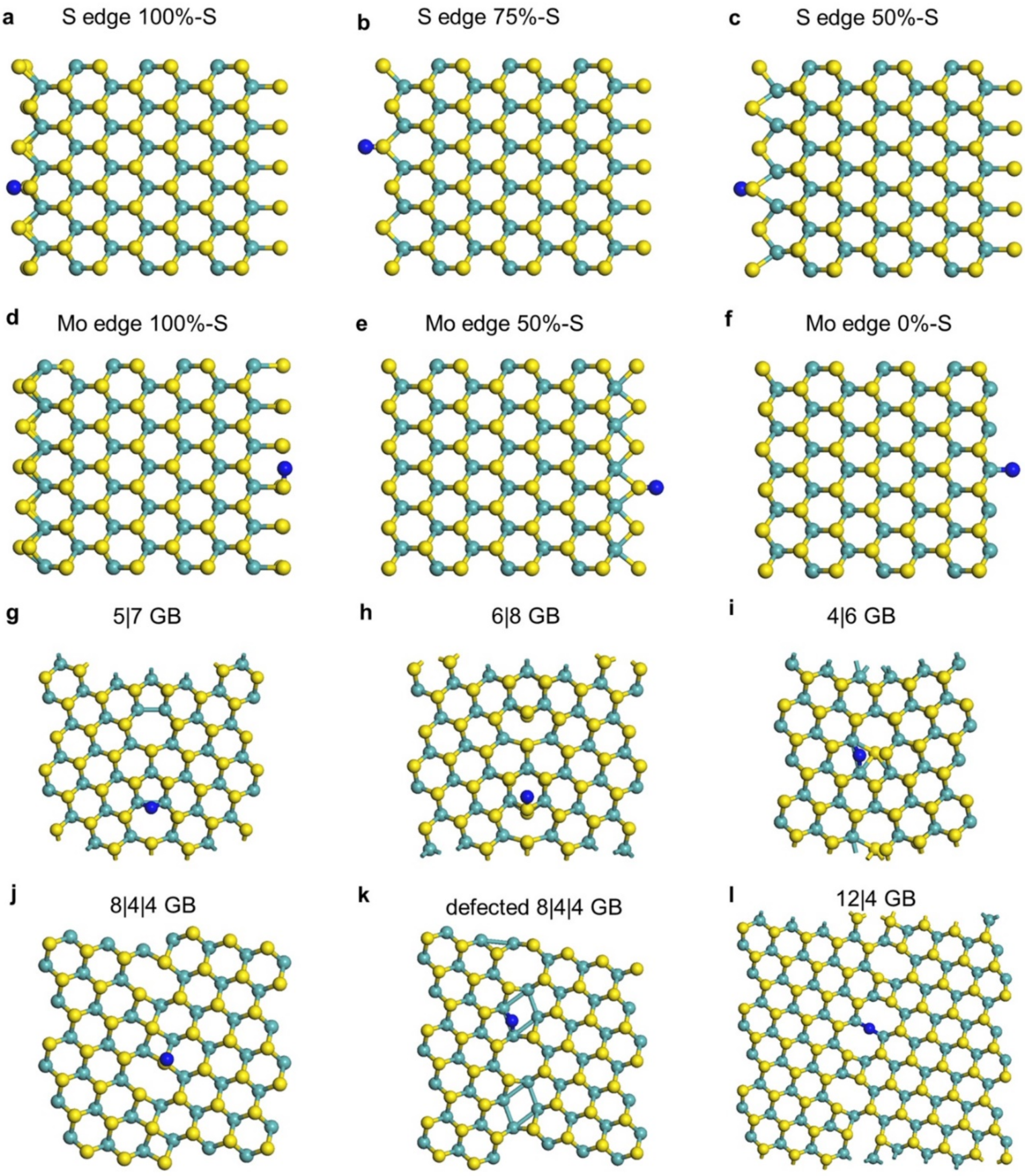

Supplementary Figure 18. Schematic of hydrogen adsorption on various atomic structures in MoS_2. (a)-(c) S edge with 100% (a), 75% (b), and 50% (c) S-passivation. (d)-(f) Mo edge with 100% (d), 50% (e), and 0% (f) S-passivation. (g) 5|7 GB. (h) 6|8 GB. (i) 4|6 GB. (j) 8|4|4 GB. (k) Defected 8|4|4 GB. (l) 12|4 GB. Green spheres represent Mo atoms, yellow spheres represent S atoms and blue spheres, H.

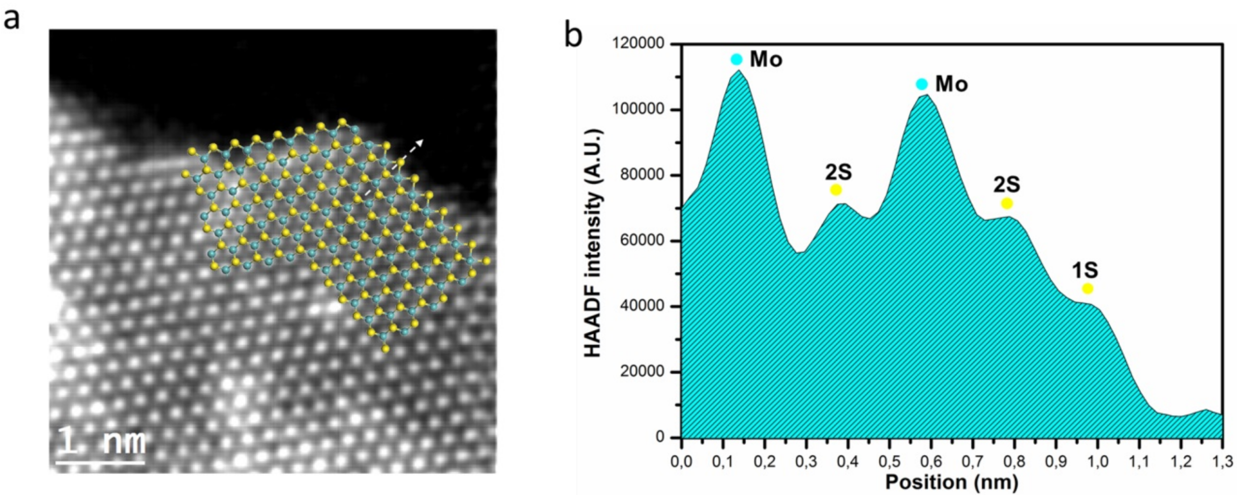

Supplementary Figure 19. Atomic resolution HAADF image of the edge of CVD-grown single-layer MoS_2. (a) Atomic resolution HAADF STEM images of the atomic structure at the edge region. (b) Profile of the atomic column intensity along the white dotted arrow direction in (a) demonstrating that the 50% S-terminated Mo edge structure dominates the edge region. It shows that the 50% S-terminated Mo edge is prevalent in our CVD-grown single-layer MoS_2 sample. Knock-on damage in electron microscopy can be reduced by lowering of the primary electron energy below its threshold energy, which is approximately 66 keV for bulk MoS_2. In our experiments, the effects of knock-on damage on the sample were drastically reduced via employing 60 keV primary energy for STEM-HAADF image acquisition, although we note that ionization damage can still induce severe beam damage, while edge and defected structures may also be less stable than the bulk, with lower knock on damage thresholds, making their observation particularly challenging[2,3].

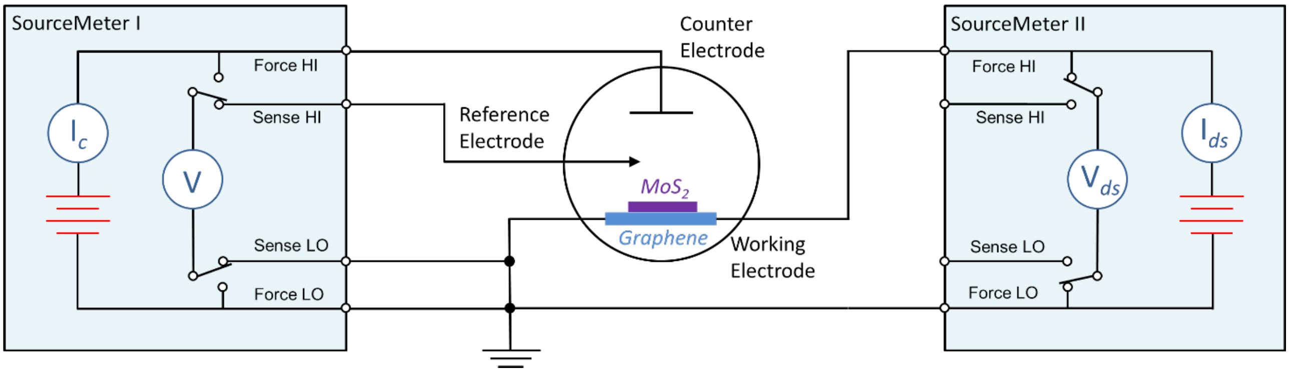

Supplementary Figure 20. Configuration of the four-electrode micro-electrochemical cell. The circuit diagram of the measurement configuration includes two source measurement units (Keithley 2450 and 2400). SMU1 (Keithley 2450) serves as the three-electrode electrochemical measurement unit, where both Low Force (Force LO) and Low Sense (Sense LO) were connected with the working electrode, and High Force (Force HI) and High Sense (Sense HI) were connected together to the counter electrode and reference electrode, respectively. The corresponding electrocatalytic current (I_c) is collected on SMU1. SMU2 (Keithley 2400) serves as the conductance measurement unit, where High Force (Force HI) and Low Force (Force LO) were connected to the electrodes on the graphene supporting layer. The corresponding conductance current (I_{ds}) is collected during the electrocatalytic process. These two measurements can be effectively carried out synchronously through GPIB and Labview interface. As a result, both the electrocatalytic signal of a MoS_2 device and the conductance signal of the graphene supporting layer in HER can be simultaneously recorded.

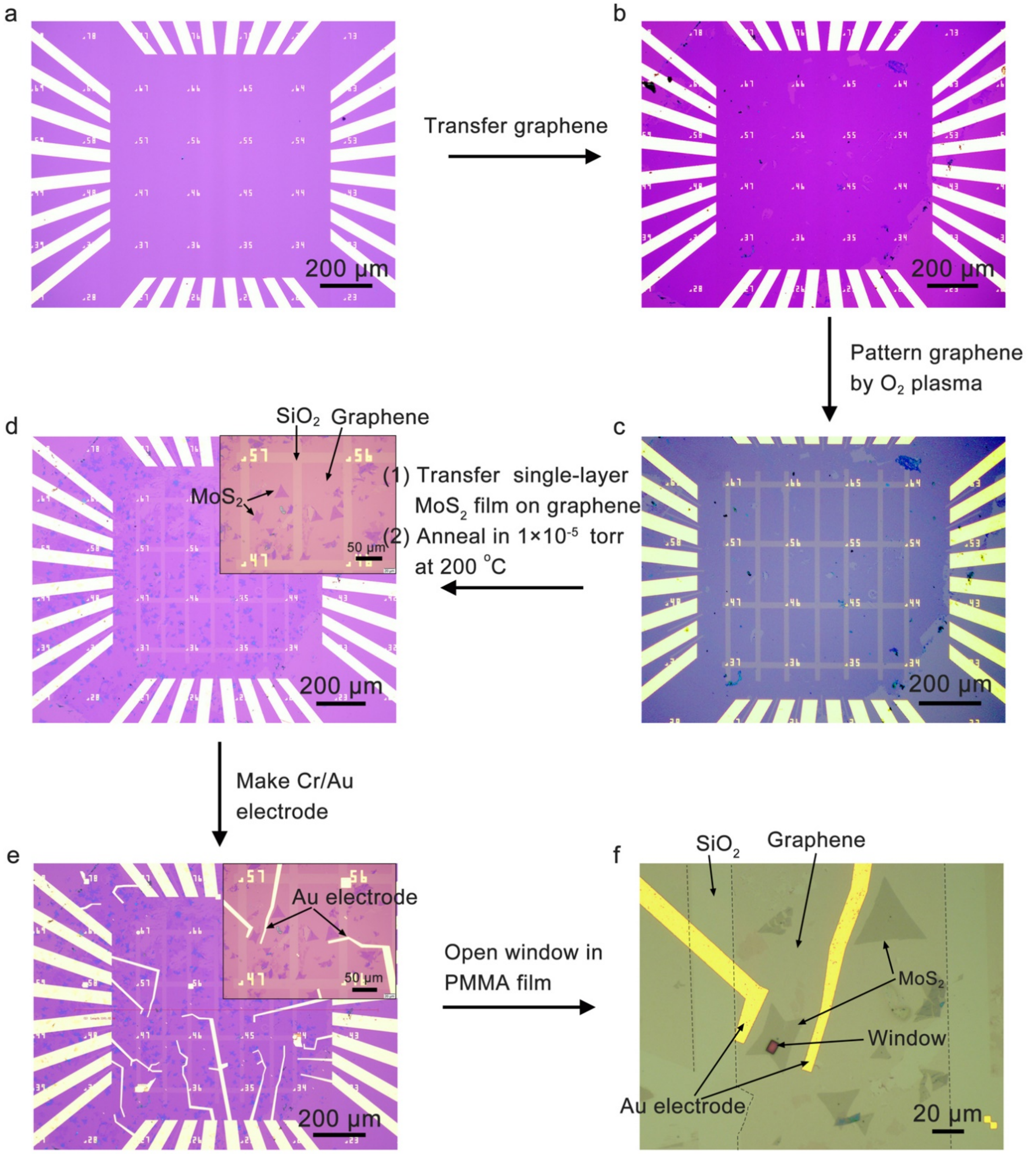

Supplementary Figure 21. Fabrication procedure for the single-layer MoS_2 microelectrode on a graphene supporting layer.

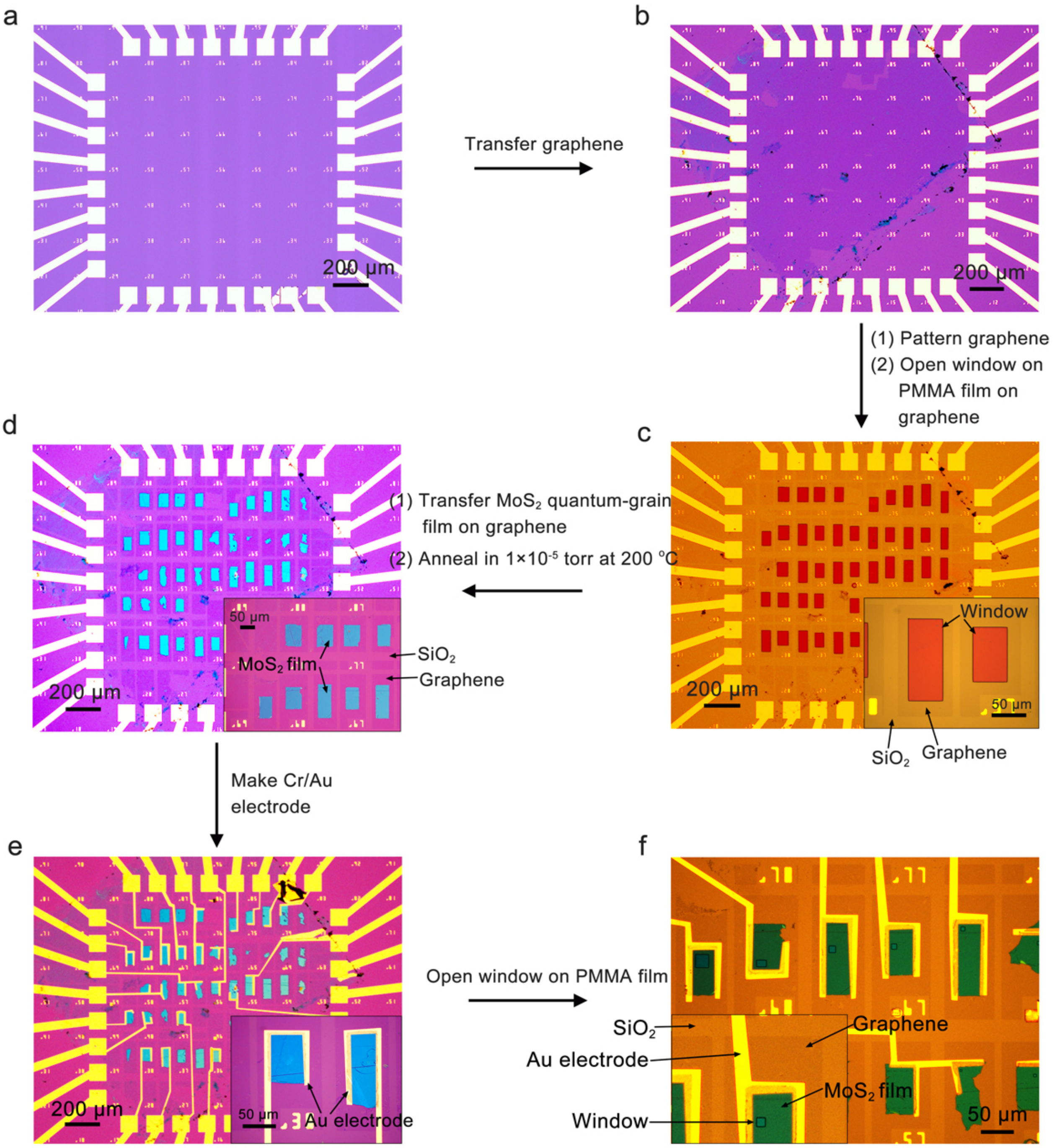

Supplementary Figure 22. Fabrication procedure of the MoS_2 nanograin film microelectrode on a graphene supporting layer (4-5 L used in this figure).

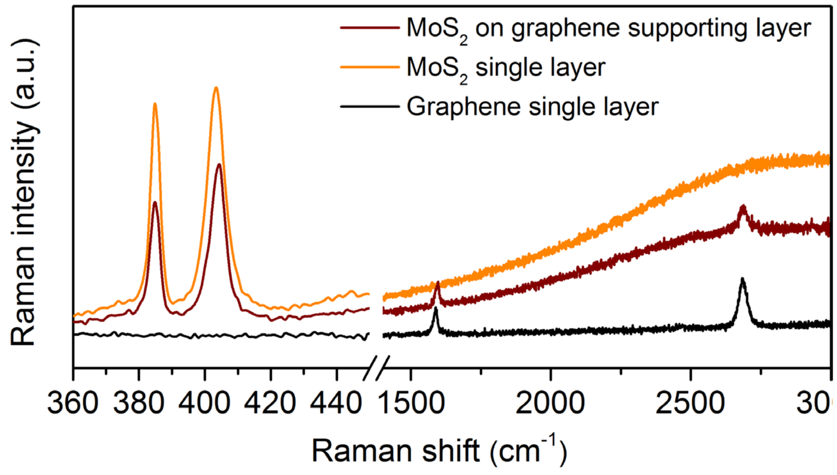

Supplementary Figure 23. Raman spectrum of single-layer graphene, single-layer MoS_2, and MoS_2 on the graphene supporting layer.

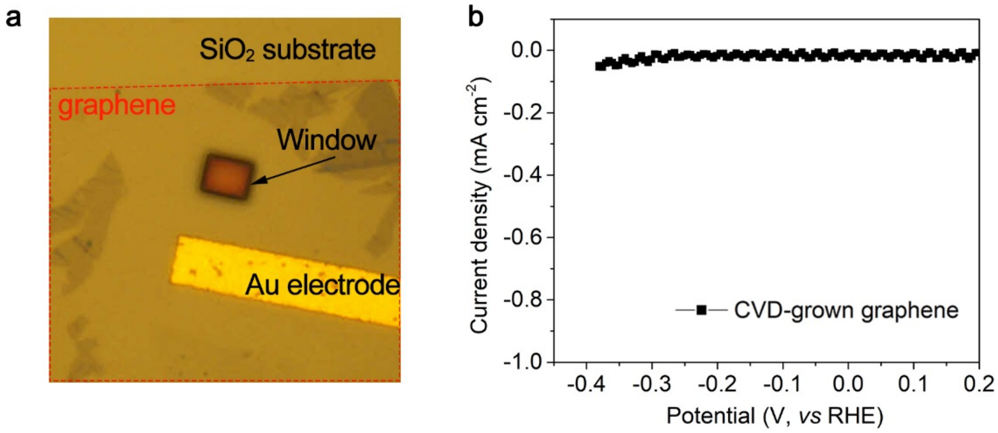

Supplementary Figure 24. HER activity of CVD-grown monolayer graphene layer. (a) Optical image of a CVD-grown monolayer graphene microelectrode. (b) Polarization curves of the current density of the graphene microelectrode. A current density of less than 0.1 mA cm^{-2} was measured, suggesting the CVD-grown graphene delivers a high quality with few defects, and can thus be considered electrochemically inert.

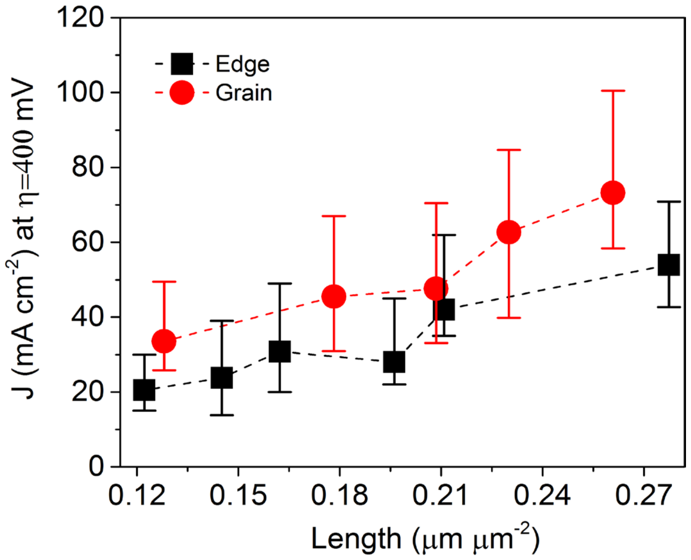

Supplementary Figure 25. Statistics of current density with catalytic active length per unit area for individual edge and GB structures in MoS_2. It is well known that comparing the exchange current density with catalytic active length per unit area is another effective way to identify 1D active site.[4,5] It shows that a single-GB structure indeed delivers a better HER-activity than a single-edge after testing hundreds of devices with various window sizes.

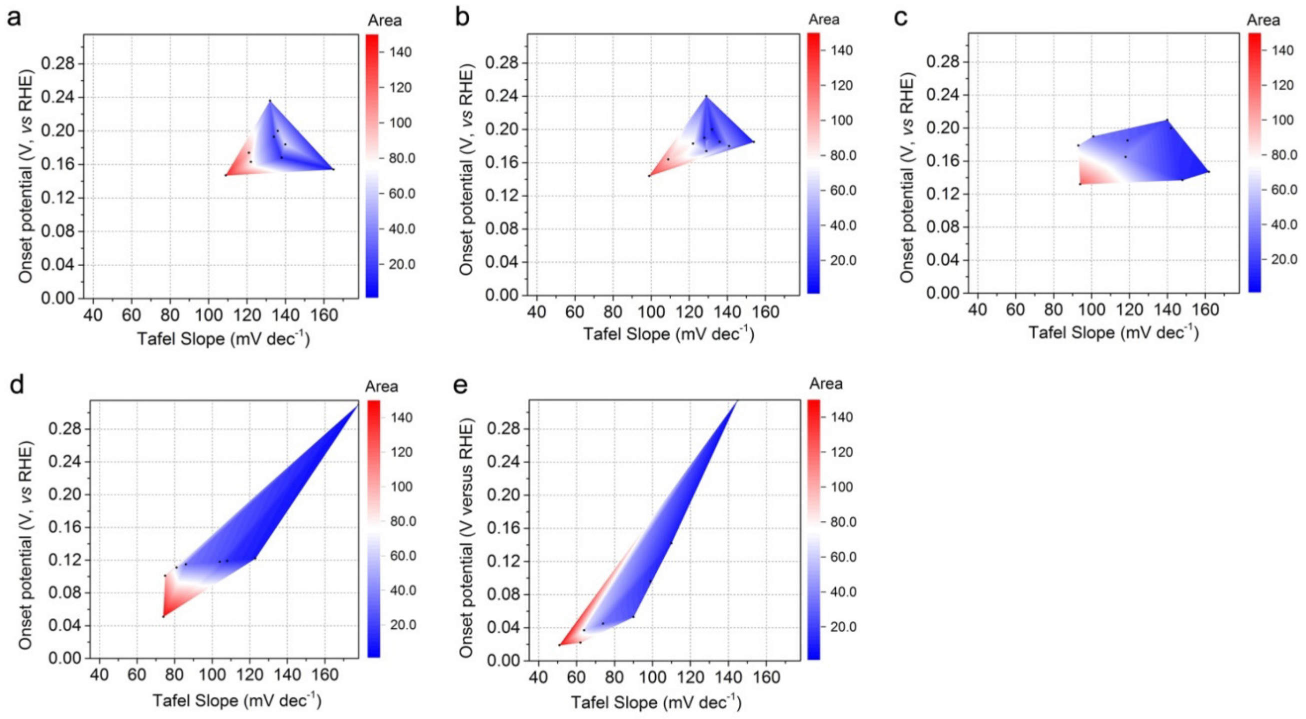

Supplementary Figure 26. Mapping HER performance for the MoS_2 basal plane (a), edge (b), GB (c), and nanograin films with 1-3 L (d) and 4-5 L (e) in size-controlled micro-electrochemical cell. In order to provide a fair comparison, the HER performance in size-controlled micro-electrochemical cells was plotted for the MoS_2 basal plane (a), edge (b), GB (c), and for different nanograin films (1-3 L in d and 4-5 L in e), in which the Tafel slope is the X axis and the onset potential is the Y axis. It is worth mentioning that the blue, low area regions in all figures are very large, indicating the instable HER performance at small window sizes.

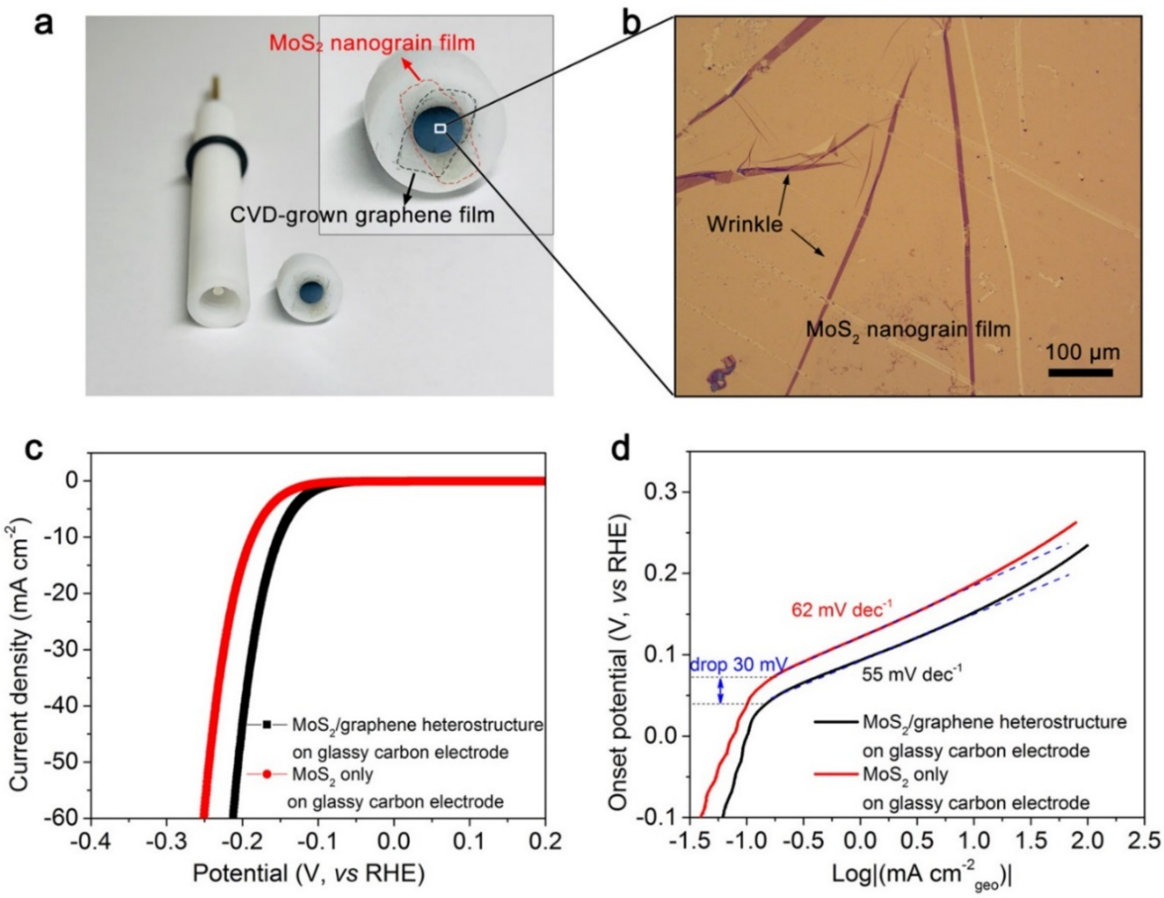

Supplementary Figure 27. The influence of the graphene supporting layer on the performance of the MoS_2 nanograin film. (a) Photograph of a MoS_2/Graphene heterostructure film on a glassy carbon electrode. The inset shows that this heterostructure film was transferred layer-by-layer on the glassy carbon electrode. (b) Opticalimages of the MoS_2/Graphene heterostructure film. (c-d) Polarization curves of the current density (c) and the corresponding Tafel plots (d) of the MoS_2 nanograin on a graphene supporting layer and glassy carbon electrode.

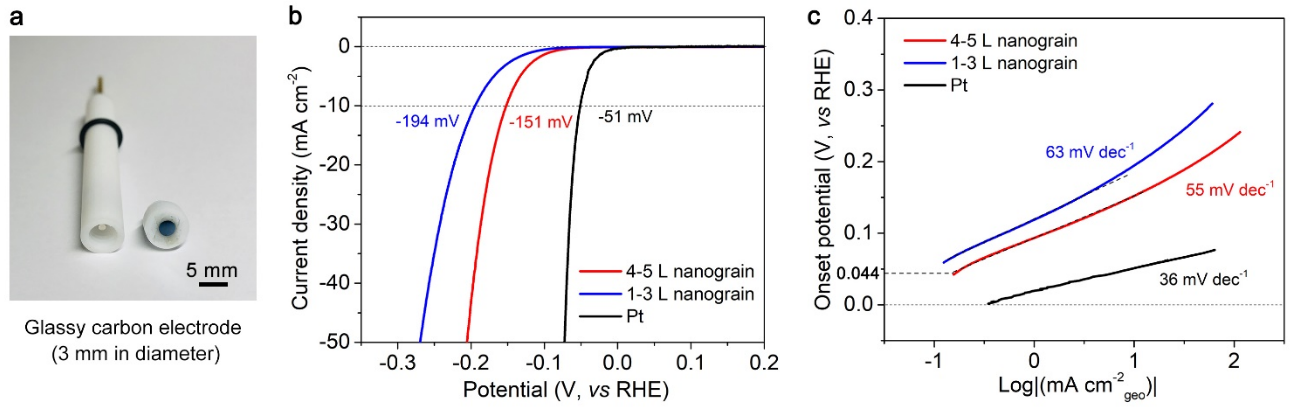

Supplementary Figure 28. HER performance of MoS_2 nanograin on a glassy carbon electrode in a macro-electrochemical cell. (a-b) Polarization curves (a) and the corresponding Tafel plots (b) of a MoS_2 1-3 L and 4-5 L nanograins film in the macro-electrochemical cell. We layer-by-layer transferred MoS_2 nanograin/graphene film on glassy carbon electrode, as shown in **a**. It can be seen from **b** and **c** that MoS_2 nanograin film showa a remarkable catalytic performance: onset potential of -44 mV and Tafel slope of 55 mV dec^{-1}, which is similar to the results measured in microcell (onset potential of -25 mV and Tafel slope of 54 mV dec^{-1}).

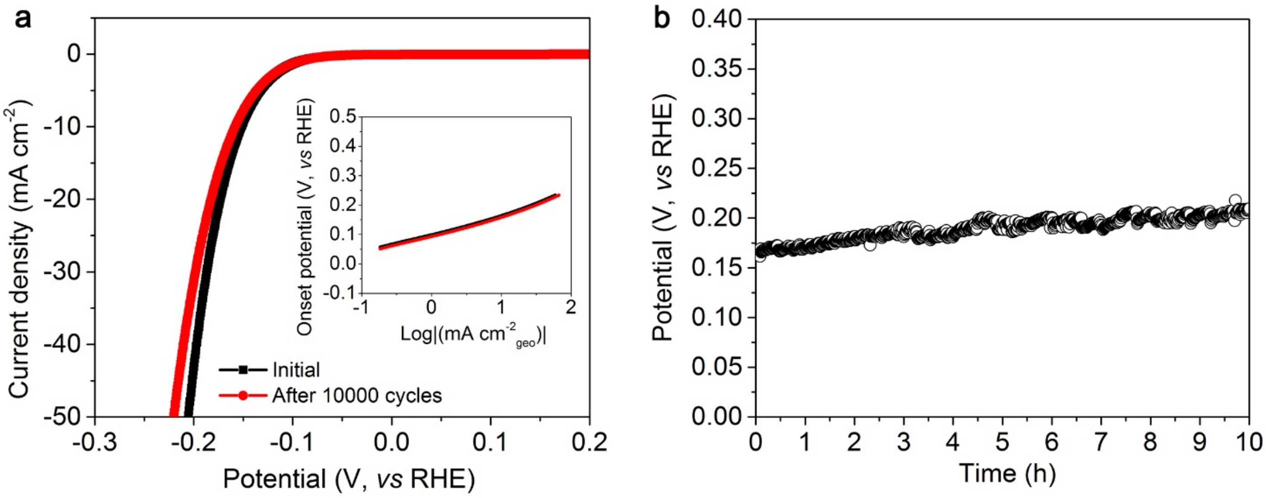

Supplementary Figure 29. HER stability test of the MoS_2 nanograin film. (a) Long-term stability test for the MoS_2 nanograin film. The polarization curves were recorded at a scan rate of 5 mV s^{-1} after 10,000 potential cycles. The current density was normalized by the geometric surface area of the electrode. (b) Time-dependent overpotential (η) curve under j = 20 mA cm^{-2} in 0.5 M H_2SO_4 aqueous solution. The MoS_2 nanograin also exhibits remarkable durability during the HER. It is seen from **a** that the polarization curve of the MoS_2 nanograin for the HER after 10,000 potential cycles is similar to the initial one, with negligible loss of the cathodic current. Furthermore, the chronopotentiometric test of overpotential (η) versus time indicates that it possesses outstanding long-term operational stability at 20 mA cm^{-2}, as shown in **b**. The overpotential exhibits a slight initial increase without significant variation over 10 h, suggesting good durability under the testing conditions.

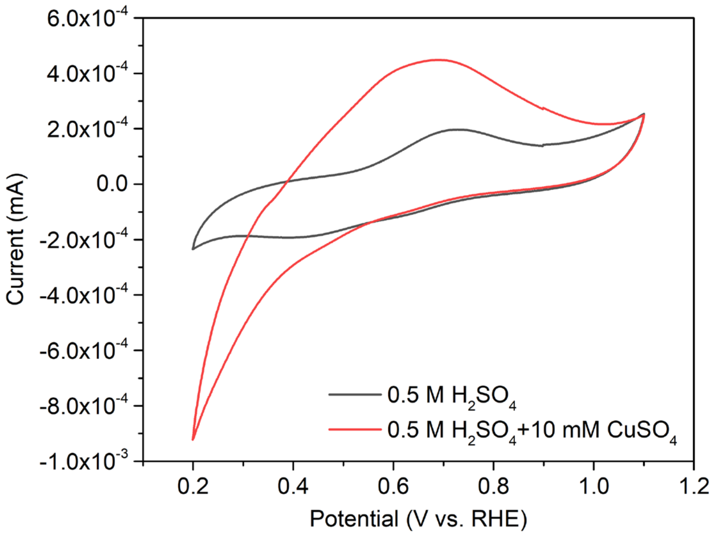

Supplementary Figure 30. Calibration of the electrochemically active surface area and the number of active sites of a MoS_2 nanograin film supported by graphene on a glassy carbon electrode through the Cu underpotential deposition (UPD) method (scan rate: 10 mV s^{-1}). Copper UPD was performed in N_2-saturated 0.5 M H_2SO_4 in the absence (black curve) and presence (red curve) of 10 mM $CuSO_4$ in accordance with the methodology used in previous reports[6-9].

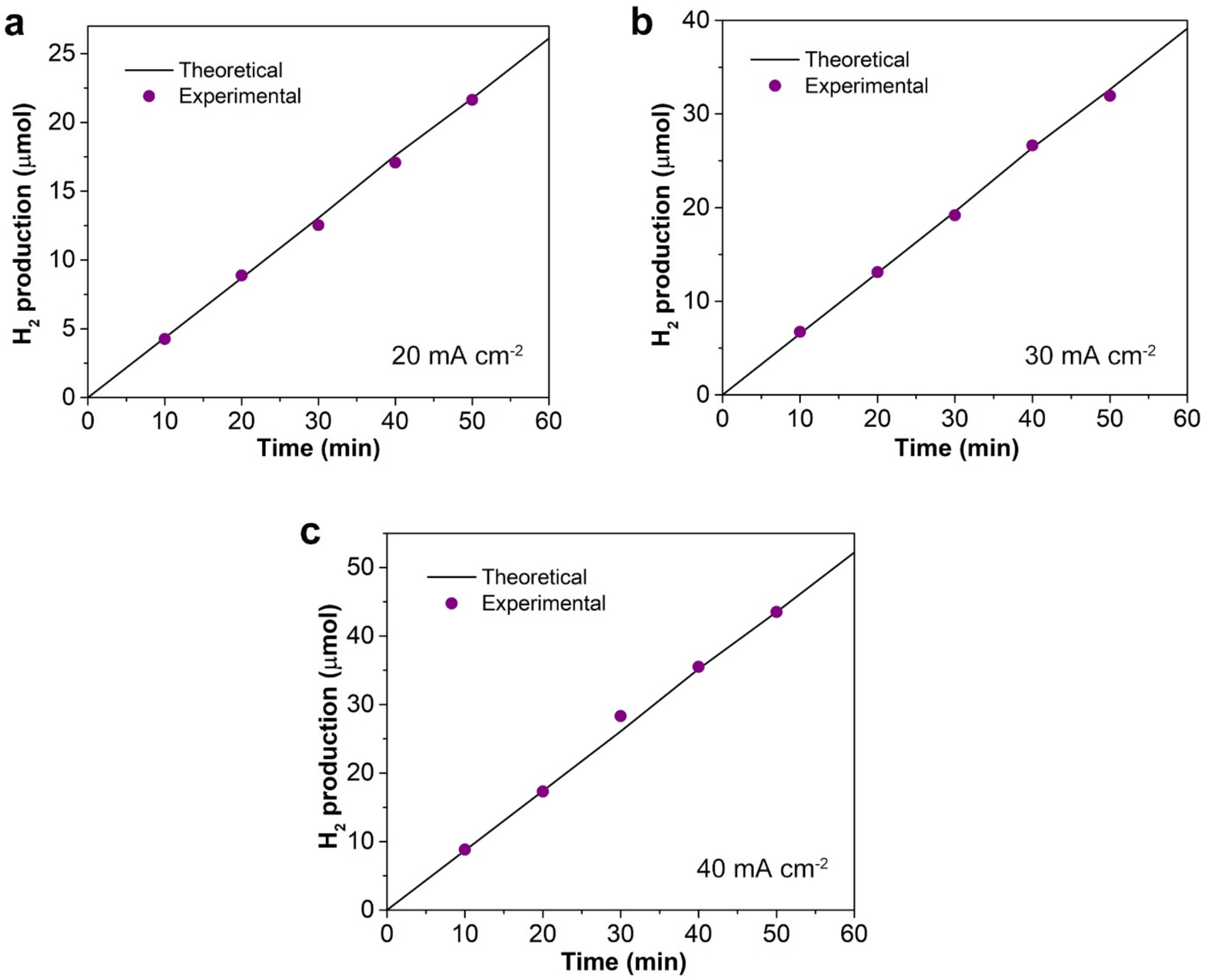

Supplementary Figure 31. Faradaic efficiencies of MoS_2 nanograin film. (a-c) The amount of H_2 theoretically calculated (black line) and experimentally measured (purple dots) versus time for MoS_2 nanograin film at varied current density: 20 mA cm^{-2} (a), 30 mA cm^{-2} (b), and 40 mA cm^{-2}, respectively. We also investigated the Faradaic efficiency of MoS_2 nanograin film for hydrogen production using a previously-reported method[10-12]. The Faradaic efficiency was calculated by comparing the measured amount of H_2 generated by cathodal electrolysis in GC setup with the calculated amount of H_2 in the electrochemical measurements, as shown in Experiment Method. We observed a good correlation between the calculated and experimental amounts of H_2 gas under varied current densities in our work. The faradaic yields of H_2 in our MoS_2 nanograin film are 98.4±2. 5%, 100.0±2.2%, and 102.0±3.6% at 20, 30 and 40 mA cm^{-2}, respectively. The results indicate a high-efficient hydrogen production (nearly 100% energy conversion) in our work.

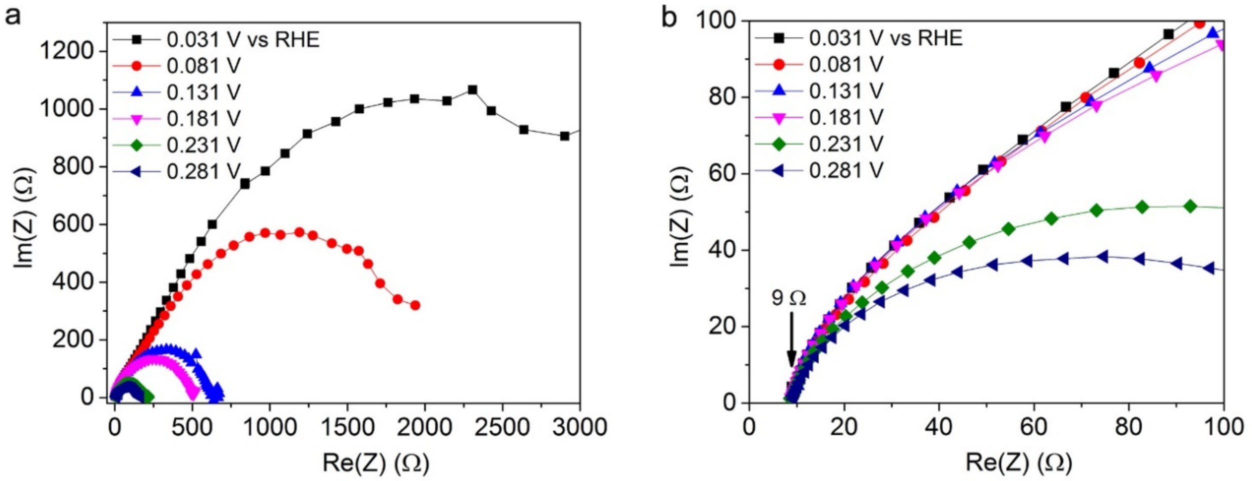

Supplementary Figure 32. Electrochemical impedance spectroscopy (EIS) measurement of MoS_2 nanograin film (a) and (b).

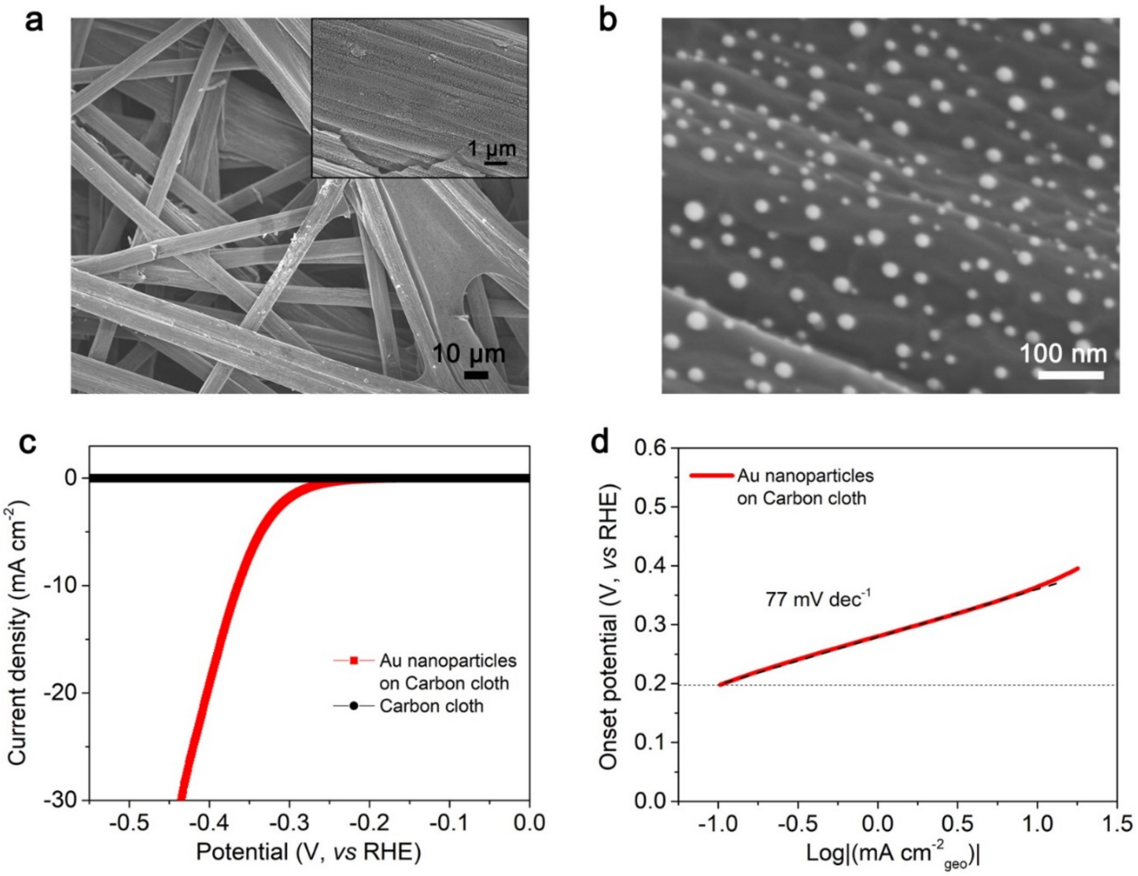

Supplementary Figure 33. Investigation of the HER activity of Au nanoparticles. (a-b) SEM images of Au nanoparticles on a carbon cloth. (c-d) Polarization curves of the current density (c) and the corresponding Tafel plots (d) of the Au nanoparticles on carbon cloth.

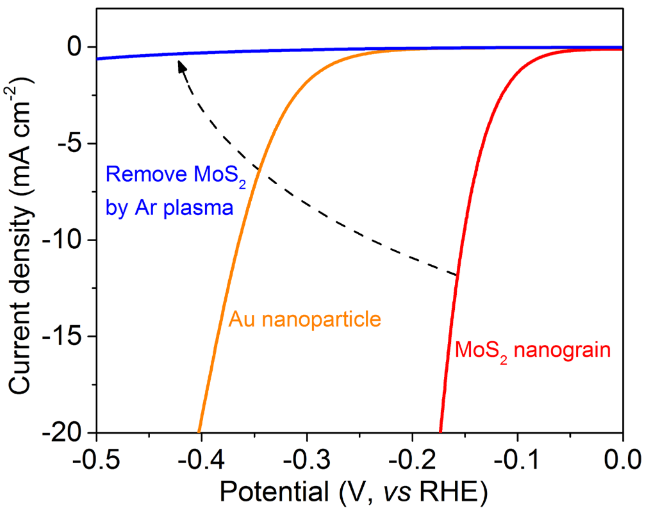

Supplementary Figure 34. HER measurement of the film after removing MoS_2 by O_2 plasma on glassy carbon electrode.

Supplementary Tables

Sample	E_{2g} Peak Position (cm^{-1})	A_{1g} Peak Position (cm^{-1})	E_{2g} FWHM (cm^{-1})	$I_{(E2g)}/I_{(A1g)}$
This work	384	405	19.0	0.34
Mechanical Exfoliation	383	404	6.9	1.42
CVD-growth	383	404	6.9	1.73

Supplementary Table 1. Raman data of the three types of MoS_2 samples from Figure 1f.

Configuration	ΔG (eV)
Basal plane	1.86082
Mo 0%	-0.44649
Mo 50%	0.56124
Mo 100%	0.24748
S 50%	0.44673
S 75%	0.92619
S 100%	-0.40284
5-7 GB	-0.23741
6-8 GB	0.13206
4-6 GB	1.00043
844 GB	0.52029
decfect 844 GB	-0.04452
12-4 GB	-0.741

Supplementary Table 2. ΔG_H of various atomic structures in MoS_2.

Catalyst	Q_{Cu} (mC)	Electrochemically active surface area (ECSA) (cm^2)	Number of active sites (× 10^{-3} mol)
MoS_2 nanograin	0.01167	0.02778	6.04664E-8

Supplementary Table 3. Evaluation of the ECSA of the MoS_2 nanograin film, in which QCu, ECSA, and the number of active sites are listed.

Supplementary Notes

Supplementary Note 1

Discussion of Raman data. The Phonon Confinement Model (PCM) is widely used to derive the grain size in polycrystalline materials from Raman spectra[13], and is suitable for grains with a size of > 5 nm. According to this model, the Raman line intensity, i.e., I(ω), at the frequency ω can be written as[14]:

$$I(\omega) = \int_0^1 \exp\left(\frac{-q^2L^2}{4}\right) \frac{d^3q}{[\omega-\omega(q)]^2+(\frac{\Gamma_0}{2})^2} \qquad \text{(Supplementary Equation 1)}$$

where q is the wave vector expressed in units of 2π/a (a is the lattice constant), Γ_0 is the half width of the Raman peak, L is the correlation length, and ω(q) is the function of Raman phonon dispersion. Note that only in a perfect lattice is L equal to d_g because there are no defects in the lattice to act as phonon scattering centers. However, in most cases, L is much smaller than d_g. L is affected by many factors[13] such as grain size, defects/impurities interspacing, the size of polytypic domains or clusters in semiconducting alloys, etc.

Prior work reported[14] in thin-film materials suggest that: when the grain size is > 100 nm, Γ will not decrease with grain size, because nearly all of the phonon dispersion arises are from defects inside the grain; in contrast, when the grain size is < 100 nm, Γ decreases with grain size because the phonon dispersion is mainly caused by GBs. As for 2D ultra-thin films, Γ will be greatly decreased due to the in-plane mode while hardly affected by out-of-plane mode. This indicates that in-plane Γ of the nanograin films should be much smaller than that in CVD-grown and mechanically-exfoliated MoS_2. According to equation (5), the intensity of the E_{2g} mode in the nanograin film is calculated to be much smaller compared to the two other types of samples. At the same time, the intensity of the A_{1g} mode is comparatively unchanged. This results in a significant lower $I_{(E2g)}/I_{(A1g)}$ ratio for the nanograin film, which is in consistent with our experimental observation. Additionally, the E_{2g} peak full width at half maximum (FWHM) of the nanograin MoS_2 film in this work is three times larger than that of CVD-grown and mechanically-exfoliated MoS_2. According to the relationship between FWHM and grain size,[14] this suggests that the grains in this work are much smaller than those in CVD-grown and mechanically-exfoliated MoS_2.

Supplementary Note 2

Fabrication procedure for the single-layer MoS_2 microelectrode on a graphene supporting layer. First, a 16 mm×16 mm SiO_2 (285 nm)/Si chip with a pre-patterned set of 32 Au contact pads was fabricated using conventional photolithography (Supplementary Figure 21a). Second, a large-scale single-layer graphene film was grown on Cu foils by CVD[15], and then transferred onto the pre-patterned chip through the conventional PMMA-assisted transfer method (Supplementary Figure 21b). Third, EBL and O_2 plasma were employed to pattern the graphene film into isolated small strips (Supplementary Figure 21c). Fourth, a single-layer MoS_2 film from the CVD growth was transferred onto the patterned graphene, and a further annealing process at 200 °C under high-vacuum conditions (1×10^{-5} torr) was conducted to optimize their interfaces (Supplementary Figure 21d). Fifth, EBL and thermal evaporation were performed to fabricate the electrodes (Cr/Au, 2 nm/60 nm) on graphene to connect the Au contact pads (Supplementary Figure 21e). Finally, a window was opened in the PMMA passivation film by EBL to expose different MoS_2 regions (such as the basal plane, edges, and GBs) for HER (Supplementary Figure 21f).

Supplementary Note 3

Fabrication procedure of the MoS_2 nanograin film microelectrode on a graphene supporting layer. First, a 16 mm×16 mm SiO_2 (285 nm)/Si chip with a set of 32 pre-patterned Au contact pads was fabricated using conventional photolithography (Supplementary Figure 22a). Second, a large-scale single-layer graphene film was grown on Cu foils by CVD[15], and then transferred onto on the pre-patterned chip through the PMMA-assisted transfer method (Supplementary Figure 22b). Third, EBL and O_2 plasma were employed to pattern the graphene film into isolated small strips. After that, a PMMA film (A7, 950 k) was spin-coated on the patterned graphene substrate as a template. Another EBL step was then used to open windows on PMMA to expose a partial graphene pattern for further transfer of the MoS_2 nanograin film onto it (Supplementary Figure 22c). Fourth, the free-standing MoS_2 nanograin film was lifted off from the as-grown SiO_2/Si substrate by PMMA-assisted transfer and KOH etching, and deposited onto the PMMA template. After removing the PMMA with acetone, the MoS_2 nanograin film was deposited on the graphene substrate with the desired size and shape (Supplementary Figure 22d). Fifth, EBL and thermal evaporation were performed to fabricate the

electrodes (Cr/Au, 2 nm/60 nm) on graphene to connect the Au contact pads (Supplementary Figure 22e). Finally, a window was opened in the PMMA passivation film by EBL to expose different regions of the MoS_2 nanograin film for HER (Supplementary Figure 22f).

Supplementary Note 4

The influence of the graphene supporting layer on the performance of the MoS2 nanograin film. In order to verify the role of the graphene layer, we made a control experiment to examine the activity and the performance of the MoS_2 nanograin with/without the graphene supporting layer on a glassy carbon electrode, as shown in Supplementary Figure 27. MoS_2/Graphene heterostructure film was fabricated through layer-by-layer transfer of CVD-grown graphene and MoS_2 nanograin films on a glassy carbon electrode (Supplementary Figure 27 a and b), in which the sample area exceeding to glassy carbon electrode was scraped off before measuments. The electrochemical measurements (Supplementary Figure 27 c and d) show that there is no considerable change in the HRE activity of either sample, suggesting that the HER activity stems mainly from the MoS_2 nanograin film, and not the graphene itself. The introduced graphene layer can improve the HER performance of MoS_2 nanograin, and a 30 mV (vs. RHE) drop in the onset potential for the MoS_2/Graphene heterostructure. This indicates that the graphene layer can facilitate electron injection into the MoS_2 due to their low-barrier band alignment[16-18], which is a strategy that is widely adopted in TMD semiconductor devices[19-21].

Supplementary Note 5

Calibration of the electrochemically active surface area and the number of active sites. The ECSA (electrochemical surface area) of the MoS_2 nanograin films was further investigated on a glassy carbon electrode, as shown in Supplementary Figure 30. The red CV curves show the signal of Cu underpotential stripping. The Cu underpotential deposition (UPD) method [6-9] has been widely applied for quantifying the number of active sites and the electrochemically active surface area (ECSA) of catalysts. In this method, the number of active sites can be calculated on the base of the UPD Cu stripping charge (Q_{Cu}, $Cu_{upd} \rightarrow Cu^{2+} + 2e^-$) using the following equation: $n = Q_{Cu}/(2F)$, where F is the Faraday constant (96485 C mol^{-1}). Assuming a value of 420 $\mu C\ cm^{-2}$ for a saturated Cu_{upd} monolayer

formation on active metal sites[8,9], the ECSA can be calibrated as: ECSA = Q_{Cu}/(420 μC cm^{-2}), and the values are shown in Supplementary Table 3.

Supplementary Note 6

Investigation of the HER activity of Au. In order to investigate the HER-activity contribution of the Au nanoparticles to our experiments, we carry out a control experiment whereby a 1 nm-thick Au layer was deposited Au onto carbon cloth, then followed exposed to the same growth condition of MoS_2 but without Mo and S sources, and finally measure its HER performance. Therefore, we can make a clear comparison conditions as for the HER performance of Au with and without MoS_2 in our experiments. Supplementary Figure 33 a and b show the morphology of the Au nanoparticles. The electrochemical measurements (Supplementary Figure 33 c and d) show that the Au nanoparticle on carbon cloth resulting sample delivers a moderate HER performance (onset potential of - 195 mV, Tafel slope of 77 mV dec^{-1}), respectively, the value of which is consistent with previous reports of Au HER-activity[22-29].

We further examined the activity of the residual Au by completely removing MoS_2 using O_2 plasma (20 W for 10 ~ 20 min etching time) and conducted HER measurement again on the remaining Au. Supplementary Figure 34 shows that the remaining Au delivers a negligible HER performance compared to original MoS_2 nanograin.

Supplementary References

1 Nanda, K. K., Sahu, S. N. & Behera, S. N. Liquid-drop model for the size-dependent melting of low-dimensional systems. *Phys. Rev. A* **66**, 013208 (2002).

2 Zhou, W. *et al.* Intrinsic Structural Defects in Monolayer Molybdenum Disulfide. *Nano Lett.* **13**, 2615-2622 (2013).

3 Hansen, L. P. *et al.* Atomic-Scale Edge Structures on Industrial-Style MoS_2 Nanocatalysts. *Angew. Chem. Int. Ed.* **50**, 10153-10156 (2011).

4 Jaramillo, T. F. *et al.* Identification of active edge sites for electrochemical H_2 evolution from MoS_2 nanocatalysts. *Science* **317**, 100-102 (2007).

5 Ji, Q. *et al.* Morphological Engineering of CVD-Grown Transition Metal Dichalcogenides for Efficient Electrochemical Hydrogen Evolution. *Adv. Mater.* **28**, 6207-6212 (2016).

6 Zheng, Y. *et al.* High Electrocatalytic Hydrogen Evolution Activity of an Anomalous Ruthenium Catalyst. *J. Am. Chem. Soc.* **138**, 16174-16181 (2016).

7 Mahmood, J. *et al.* An efficient and pH-universal ruthenium-based catalyst for the hydrogen evolution reaction. *Nature Nanotechnology* **12**, 441 (2017).

8 Green, C. L. & Kucernak, A. Determination of the Platinum and Ruthenium Surface Areas in Platinum−Ruthenium Alloy Electrocatalysts by Underpotential Deposition of Copper. I. Unsupported Catalysts. *J. Phys. Chem. B* **106**, 1036-1047 (2002).

9 Colmenares, L., Jusys, Z. & Behm, R. J. Electrochemical Surface Characterization and O_2 Reduction Kinetics of Se Surface-Modified Ru Nanoparticle-Based $RuSe_y$/C Catalysts. *Langmuir* **22**, 10437-10445 (2006).

10 Zhou, H. *et al.* Efficient hydrogen evolution by ternary molybdenum sulfoselenide particles on self-standing porous nickel diselenide foam. *Nat. Commun.* **7**, 12765 (2016).

11 Kleingardner, J. G., Kandemir, B. & Bren, K. L. Hydrogen Evolution from Neutral Water under Aerobic Conditions Catalyzed by Cobalt Microperoxidase-11. *J. Am. Chem. Soc.* **136**, 4-7 (2014).

12 Beyene, B. B., Mane, S. B. & Hung, C.-H. Highly efficient electrocatalytic hydrogen evolution from neutral aqueous solution by a water-soluble anionic cobalt(ii) porphyrin. *Chem. Commun.* **51**, 15067-15070 (2015).

13 Gouadec, G. & Colomban, P. Raman Spectroscopy of nanomaterials: How spectra relate to disorder, particle size and mechanical properties. *Prog. Cryst. Growth Charact. Mater.* **53**, 1-56 (2007).

14 Kosacki, I., Suzuki, T., Anderson, H. U. & Colomban, P. Raman scattering and lattice defects in nanocrystalline CeO_2 thin films. *Solid State Ionics* **149**, 99-105 (2002).

15 Li, X. *et al.* Large-Area Synthesis of High-Quality and Uniform Graphene Films on Copper Foils. *Science* **324**, 1312-1314 (2009).

16 Liu, Y. *et al.* Toward Barrier Free Contact to Molybdenum Disulfide Using Graphene Electrodes. *Nano Lett.* **15**, 3030-3034 (2015).

17 Bertolazzi, S., Krasnozhon, D. & Kis, A. Nonvolatile Memory Cells Based on MoS_2/Graphene Heterostructures. *ACS Nano* **7**, 3246-3252 (2013).

18 Allain, A., Kang, J., Banerjee, K. & Kis, A. Electrical contacts to two-dimensional semiconductors. *Nat. Mater.* **14**, 1195 (2015).

19 Li, Y. *et al.* MoS_2 nanoparticles grown on graphene: an advanced catalyst for the hydrogen evolution reaction. *J. Am. Chem. Soc.* **133**, 7296-7299 (2011).

20 Mao, S. *et al.* Perpendicularly Oriented $MoSe_2$/Graphene Nanosheets as Advanced Electrocatalysts for Hydrogen Evolution. *Small* **11**, 414-419 (2015).

21 Voiry, D., Yang, J. & Chhowalla, M. Recent strategies for improving the catalytic activity of 2D TMD nanosheets toward the hydrogen evolution reaction. *Adv. Mater.* **28**, 6197-6206 (2016).

22 Li, H. *et al.* Activating and optimizing MoS_2 basal planes for hydrogen evolution through the formation of strained sulphur vacancies. *Nat. Mater.* **15**, 48-53 (2016).

23 Li, H. *et al.* Kinetic Study of Hydrogen Evolution Reaction over Strained MoS_2 with Sulfur Vacancies Using Scanning Electrochemical Microscopy. *J. Am. Chem. Soc.* **138**, 5123-5129 (2016).

24 Shin, S., Jin, Z., Kwon, D. H., Bose, R. & Min, Y.-S. High Turnover Frequency of Hydrogen Evolution Reaction on Amorphous MoS_2 Thin Film Directly Grown by Atomic Layer Deposition. *Langmuir* **31**, 1196-1202 (2015).

25 Zhang, Y. *et al.* Chemical vapor deposition of monolayer WS_2 nanosheets on Au foils toward direct application in hydrogen evolution. *Nano Res.* **8**, 2881-2890 (2015).

26 Shi, J. *et al.* Controllable Growth and Transfer of Monolayer MoS_2 on Au Foils and Its Potential Application in Hydrogen Evolution Reaction. *ACS Nano* **8**, 10196-10204 (2014).

27 Zhang, Y. *et al.* Dendritic, Transferable, Strictly Monolayer MoS_2 Flakes Synthesized on $SrTiO_3$ Single Crystals for Efficient Electrocatalytic Applications. *ACS Nano* **8**, 8617-8624 (2014).

28 Zhao, S. *et al.* Atomically Precise Gold Nanoclusters Accelerate Hydrogen Evolution over MoS_2 Nanosheets: The Dual Interfacial Effect. *Small* **13**, 1701519 (2017).

29 Zhang, J. *et al.* Molybdenum disulfide and Au ultrasmall nanohybrids as highly active electrocatalysts for hydrogen evolution reaction. *J. Mater. Chem. A* **5**, 4122-4128 (2017).
